# Targeting mitochondrial quality control: new therapeutic strategies for major diseases

**DOI:** 10.1186/s40779-024-00556-1

**Published:** 2024-08-21

**Authors:** Wei-Long Hong, He Huang, Xue Zeng, Chen-Yang Duan

**Affiliations:** Department of Anesthesiology, the Second Affiliated Hospital of Chongqing Medical University, Chongqing, 400010 China

**Keywords:** Major diseases, Mitochondrial quality control, Mitochondrial targeted therapy

## Abstract

Mitochondria play a crucial role in maintaining the normal physiological state of cells. Hence, ensuring mitochondrial quality control is imperative for the prevention and treatment of numerous diseases. Previous reviews on this topic have however been inconsistencies and lack of systematic organization. Therefore, this review aims to provide a comprehensive and systematic overview of mitochondrial quality control and explore the possibility of targeting the same for the treatment of major diseases. This review systematically summarizes three fundamental characteristics of mitochondrial quality control, including mitochondrial morphology and dynamics, function and metabolism, and protein expression and regulation. It also extensively examines how imbalances in mitochondrial quality are linked to major diseases, such as ischemia-hypoxia, inflammatory disorders, viral infections, metabolic dysregulations, degenerative conditions, and tumors. Additionally, the review explores innovative approaches to target mitochondrial quality control, including using small molecule drugs that regulate critical steps in maintaining mitochondrial quality, nanomolecular materials designed for precise targeting of mitochondria, and novel cellular therapies, such as vesicle therapy and mitochondrial transplantation. This review offers a novel perspective on comprehending the shared mechanisms underlying the occurrence and progression of major diseases and provides theoretical support and practical guidance for the clinical implementation of innovative therapeutic strategies that target mitochondrial quality control for treating major diseases.

## Background

Mitochondria are widely recognized as the powerhouses of the cell, being integral to energy production and cellular health. Their functions go beyond simply energy generation, encompassing critical aspects of cellular homeostasis and signaling. Recent advancements in mitochondrial research have emphasized their importance in various diseases, highlighting the necessity of targeting mitochondrial quality control for therapeutic interventions.

The field of mitochondrial quality control has been marked by significant breakthroughs, including an improved understanding of mitochondrial dynamics and their metabolic implications, elucidation of mechanisms for maintaining mitochondrial DNA (mtDNA), and the development of innovative techniques like mitochondrial transplantation and targeted therapy. These strides have deepened our comprehension of mitochondrial pathophysiology, including its role in aging, acute ailments such as shock and sepsis, and chronic diseases, such as cancer and diabetes.

Previous reviews on mitochondrial quality control lacked depth and breadth. Therefore, this review offers a comprehensive and systematic analysis of mitochondrial quality control, addressing gaps in previous works, particularly in integrating mitochondrial function within the broader context of cellular health and exploring its therapeutic potential.

## Basic characteristics of mitochondrial quality control

### Morphology and dynamics

Mitochondrial morphology and dynamics, also known as the organelle quality system, are primarily influenced by four main processes, namely mitochondrial fission, fusion, biogenesis, and mitophagy. These processes are crucial for maintaining mitochondrial homeostasis and cellular adaptability under physiological conditions. Mitochondrial fission helps to regulate the number of mitochondria, ensuring cellular energy production and metabolic balance. It is primarily achieved by the binding of dynamin-related protein 1 (DRP1) to fission mitochondrial 1 (FIS1; a mitochondrial membrane receptor protein), mitochondrial fission factor, and mitochondrial dynamics protein of 49/51 kD, leading to cleavage of the mitochondrial membrane at mitochondrial fission sites [1–4]. Mitochondrial fusion contributes to maintaining cellular integrity and is categorized into outer and inner mitochondrial membrane fusion, mediated by mitofusin (MFN) 1, MFN2, and optic atrophy 1 (OPA1), respectively [5, 6]. Mitochondrial biogenesis involves the generation of new mitochondria within cells. Peroxisome proliferator-activated receptor gamma coactivator-1α (PGC-1α) serves as a key regulator of the mitochondrial biogenesis process by activating various transcription factors, including nuclear respiratory factor-1 and -2, peroxisome proliferator-activated receptor, mitochondrial transcription factor A (TFAM), and estrogen-related receptor α, to enhance the transcription of genes involved in this process [7]. Mitophagy allows for selective quality control of mitochondria, ensuring effective cellular responses to adverse environmental conditions. When mitochondria are damaged (as indicated by reduced membrane potential), phosphatase and tensin homolog-induced kinase 1 (PINK1) recruits and activates the E3 ubiquitin ligase, parkin RBR E3 ubiquitin-protein ligase (PARKIN) on the outer mitochondrial membrane, leading to the ubiquitination of mitochondrial proteins. These ubiquitinated proteins then bind to p62 and facilitate their recognition and clearance by LC3-labeled autophagosomes [2].

### Function and metabolism

Mitochondria serve as the energy factory and metabolic hub of cells, providing energy and essential metabolites for cellular activities and maintaining homeostasis through various functions. These include oxidative phosphorylation (OXPHOS), generation of reactive oxygen species (ROS), regulation of calcium ions, control of mitochondrial membrane potential (MMP), and mitochondrial permeability transition through the mitochondrial permeability transition pore (mPTP). In addition, mitochondria are involved in metabolic pathways such as fatty acid β-oxidation, the tricarboxylic acid (TCA) cycle, the urea cycle, phospholipid synthesis, and ketogenesis.

OXPHOS is the process by which complexes I – V on the inner mitochondrial membrane generate a proton gradient through the electron transport chain (ETC) to drive ATP synthesis [8]. ROS, as byproducts of OXPHOS, play a role in cellular signal transduction at normal levels; however, elevated levels of ROS cause oxidative stress damage [9]. Calcium ion regulation is mainly controlled by channels on the mitochondrial membrane, such as the mitochondrial calcium uniporter (MCU) and Na^+^/Ca^2+^-Li^+^ exchanger, which coordinate the uptake and release of calcium ions, maintaining mitochondrial calcium homeostasis [10]. MMP refers to the potential difference across the inner mitochondrial membrane. The mitochondrial uncoupling protein (UCP) on the inner mitochondrial membrane can uncouple MMP and ATP synthesis by promoting the transport of protons across the mitochondrial membrane, thereby affecting energy production efficiency [11]. The mPTP, composed of several proteins including adenosine nucleotide translocase, cyclophilin D (CYPD), and voltage-dependent anion channel (VDAC), is a multi-protein channel spanning the inner and outer mitochondrial membranes [12]. Opening of the mPTP channel leads to the dissipation of MMP and release of mitochondrial calcium ions and apoptotic factors, thereby influencing cell survival and apoptosis [13].

Fatty acid β-oxidation is an energy-releasing process in which fatty acids are sequentially broken down by enzymes in the mitochondria, resulting in the production of acetyl-CoA and ketone bodies. Acetyl-CoA then enters the TCA cycle and is converted into carbon dioxide and the high-energy electron carrier’s nicotinamide adenine dinucleotide hydrogen (NADH) and flavin adenine dinucleotide hydrogen, which participate in ATP production. The urea cycle is a metabolic process that occurs between the mitochondria and cytoplasm. The mitochondrial enzymes carbamoyl phosphate synthetase 1 and ornithine transcarbamylase are involved in this cycle, converting excess ammonia into urea to maintain nitrogen balance. Additionally, mitochondria are responsible for synthesizing phospholipids and maintaining the stability of mitochondrial membrane structure and function [14]. Moreover, under specific metabolic conditions, mitochondria synthesize ketone bodies as alternative energy sources for cells, particularly during prolonged fasting or hypoglycemia, which constitutes an additional biological function [15].

### Protein expression and regulation

Mitochondria contain 13 proteins encoded by mtDNA and over 1200 proteins encoded by nuclear DNA that are synthesized in the cytoplasm and targeted to the mitochondria. The folding and localization of these proteins rely on a series of mitochondrial protein quality control systems (also called the molecular quality control systems), including mitochondrial-associated degradation (MAD), the ubiquitin–proteasome system (UPS), and mitochondrial proteases [16]. For instance, valosin-containing protein in the MAD system hydrolyzes aberrant proteins on the mitochondrial membrane, promotes protein precursor ubiquitination, and participates in mitochondrial-associated ferroptosis [17] and apoptosis [18]. S-phase kinase-associated protein 2 (SKP2) in the UPS promotes ubiquitination and proteasomal degradation of misfolded proteins on the outer mitochondrial membrane [16, 19]. In the mitochondrial protease system, at least 45 mitochondrial proteases are known, with 23 being exclusively localized in the mitochondria [20].

Within the mitochondrial matrix, ATP-dependent proteases, such as mitochondrial Lon peptidase 1 (LONP1) and caseinolytic protease X and protease P complex (CLPXP), maintain mitochondrial homeostasis by eliminating damaged or misfolded proteins [20]. Additionally, mitochondria can participate in the regulation of mitochondrial protein expression through a “sensing–integration–signal transduction” pathway, in which mitochondria capture signals such as those of cofactors and mitochondrial antiviral-signaling protein (MAVS), and integrate them through processes such as fission and fusion, followed by transmission to hormone receptors (e.g., glucocorticoid receptor, estrogen receptor, and androgen receptor). Activated hormone receptors then bind to mtDNA or nuclear DNA, affecting the transcriptional expression of mitochondria-related genes [21].

The mitochondrial unfolded protein response (mtUPR), a mitochondrial stress response, is a transcriptional activation program initiated by mitochondria to regulate nuclear-encoded mitochondrial chaperones and proteases [22]. Serving as a retrograde signaling pathway from the mitochondria to the nucleus, the mtUPR facilitates communication between the two. When the mitochondrial matrix accumulates a substantial amount of unfolded, misfolded, and dysfunctional proteins under various stress conditions, the mtUPR increases the expression of mitochondrial chaperones [heat shock protein (HSP) 40/60/70 family members] and proteases (LONP1, CLPXP, and HtrA serine peptidase 2), thereby promoting the restoration of mitochondrial protein homeostasis [23].

## Mitochondrial quality imbalances: specific manifestations in major diseases

**Fig. 1:**
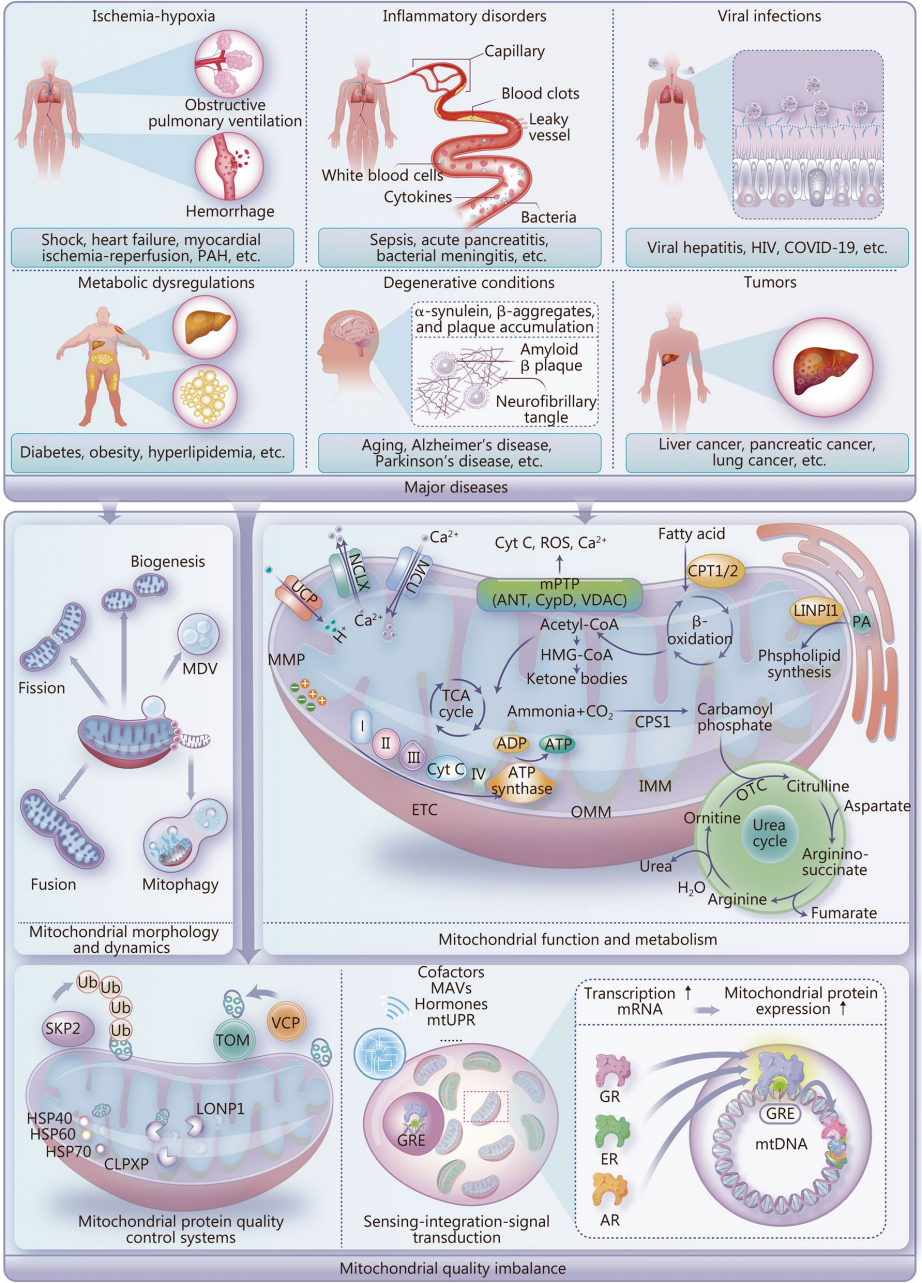
Mitochondrial quality control in the context of major diseases. Specific manifestations of major diseases, including ischemia-hypoxia, inflammatory disorders, viral infections, metabolic dysregulations, and degenerative conditions and tumors. Abnormalities in key aspects (e.g., mitochondrial morphology and dynamics, function and metabolism, and protein expression) impact the occurrence and development of various major diseases. MDV mitochondria-derived vesicle, HIV human immunodeficiency virus, COVID-19 coronavirus disease 2019, UCP uncoupling protein, NCLX mitochondrial Na^+^/Ca^2+^ exchanger, MCU mitochondrial calcium uniporter, MMP mitochondrial membrane potential, Cyt C cytochrome C, ROS reactive oxygen species, mPTP mitochondrial permeability transition pore, ANT adenosine nucleotide translocase, CYPD cyclophilin D, VDAC voltage-dependent anion channel, CPT carnitine palmitoyltransferase, CoA coenzyme A, LIPIN1 lipin 1, PA phosphatidic acid, TCA tricarboxylic acid, ADP adenosine diphosphate, ATP adenosine triphosphate, CPS1 carbamoyl-phosphate synthase 1, OMM outer mitochondrial membrane, IMM inner mitochondrial membrane, OTC ornithine transcarbamylase, ETC electron transport chain, SKP2 S-phase kinase associated protein 2, TOM translocase of outer mitochondrial membrane, VCP valosin containing protein, HSP heat shock protein, CLPXP Caseinolytic protease X and protease P complex, LONP Lon peptidase, MAV mitochondrial antiviral-signaling protein, mtUPR mitochondrial unfolded protein response, mRNA messenger RNA, GR glucocorticoid receptor, ER estrogen receptor, AR androgen receptor, GRE glucocorticoid response element (used here as an example for other gene regulatory elements), PAH pulmonary arterial hypertension, mtDNA mitochondrial DNA

Mitochondrial quality imbalance is a common characteristic in many major diseases, involving abnormalities in mitochondrial morphology and dynamics, function and metabolism, and protein expression. These impact the occurrence and development of various major diseases such as ischemia-hypoxia, inflammatory disorders, viral infections, metabolic dysregulations, and degenerative conditions, as well as tumors (Fig. 1). However, the specific manifestations and underlying pathological mechanisms of mitochondrial quality imbalance differ across diseases. Therefore, understanding the specific effects of mitochondrial quality imbalance in different disease types provides deep insights into disease progression (Table 1 [1, 23–76]). This knowledge is important for the development of mitochondria-targeted therapeutic strategies and the establishment of assessment and monitoring systems for mitochondrial quality in disease models.

**Table 1:** Changes in mitochondrial dynamics in major diseases

Classification	Diseases	Classification	Fission	Fusion	Biogenesis	Mitophagy	Reference
Ischemia-hypoxia	Shock		↑	-	-	↓	[1]
	Heart failure		↑	↓	↓	↓	[24]
	Myocardial ischemia–reperfusion		↑	↓	↓	↓	[25–27]
	Pulmonary arterial hypertension		↑	↓	↓	↑	[28–32]
Inflammatory disorders	Sepsis		↑	↓	↓	↓	[33]
	Bacterial meningitis		/	/	/	/	
	Acute pancreatitis	PC	↑	↓	/	↓	[34, 35]
		PM	/	/	↑	/	[36]
Viral infections	HBV		↑	↓	↑	↑	[37–40]
	HCV		↑	↓	/	↓	[41, 42]
	HIV	ENV	↓	↑	/	/	[43]
		TAT	↑	↓	/	↓	[44, 45]
		VPR	↓	↓	/	/	[46]
	COVID-19		↑	↓	/	↓	[47–49]
Metabolic dysregulations	Diabetes		↑	↓	↓	↓	[50–53]
	Obesity	Hepatocytes	↑	↓	-	↓	[54, 55]
		Adipocytes	/	/	↓	↓	[56]
	Hyperlipidemia		↑	↓	↓	↓ or -	[57–60]
Degenerative conditions	Aging		↑ or -	↓	↓	↓	[61–65]
	Alzheimer’s disease		↑	↓	↓	↓	[66–70]
	Parkinson’s disease		↑	↓	↓	↓	[71–74]
Tumors	Hepatocellular carcinoma		↑	↓ or -	↑	↑	[75–77]

“↑” indicate increase, “↓” indicate decrease, “-” indicate no obvious change, “/” indicate not reported. *PC* pancreatic cells, *PM* peritoneal macrophages, *HBV* hepatitis B virus, *HCV* hepatitis C virus, *HIV* human immunodeficiency virus, *HBX* HBV X protein, *COVID-19* coronavirus disease 2019, *ENV* envelope protein, *TAT* transactivator of transcription, *VPR* viral protein R

### Manifestations of mitochondrial quality imbalance in ischemia-hypoxia

Ischemia-hypoxia is the result of insufficient blood or oxygen supply to tissues and organs, leading to damage. As mitochondria are the primary site of aerobic respiration and energy production in cells, the impact of ischemic-hypoxic conditions on mitochondrial quality is direct and significant. Common ischemic-hypoxic diseases include shock, heart failure, myocardial ischemia–reperfusion, and pulmonary arterial hypertension (PAH).

#### Shock

##### Mitochondrial morphology and dynamics

In hemorrhagic shock, mitochondria primarily exhibit excessive fission due to the increased translocation of activated DRP1 from the cytoplasm to the mitochondria, leading to division. However, mitochondrial autophagy is hindered. Although mitochondrial autophagosome formation increases, autophagosome to autolysosome conversion was obstructed, resulting in ineffective clearance of damaged mitochondria [1]. Conversely, mitochondrial fusion and biogenesis remain relatively unchanged.

##### Mitochondrial function and metabolism

The occurrence of mitochondrial dysfunctions, such as decreased activity of mitochondrial complex I, is commonly observed during hemorrhagic shock, resulting in reduced ATP production [78], decreased MMP, increased ROS generation, calcium overload, excessive opening of the mPTP channel, and other mitochondrial functional impairments [12]. Additionally, abnormalities in lactate levels, depletion of phosphatidylcholine, and reduced activity of succinate dehydrogenase in the TCA cycle occur, indicating mitochondrial metabolic disturbances. The accumulation of ROS in intestinal epithelial cells during shock leads to dysbiosis of gut microbiota and inhibits the production of short-chain fatty acids [79]. In the early stages of hemorrhagic shock, choline can be generated on the mitochondrial phospholipid membrane, reversing hemorrhagic hypotension. However, as hemorrhagic shock progresses, phosphatidylcholine depletion occurs [80]. Owing to the higher metabolic demands of cardiac myocytes than other cells, mitochondrial calcium overload in cardiac shock is more severe, emphasizing the crucial role of enhancing cardiac mitochondrial metabolism in the treatment of this condition [81–83]. In septic shock, ATP depletion leads to the utilization of lactate as an alternative substrate for maintaining the hypometabolic activity of cardiac tissue [84]. When lactate depletion occurs, the lack of energy substrates rapidly precipitates cardiovascular failure [85].

##### Mitochondrial protein expression and regulation

Under shock conditions, changes occur in signaling pathways, including the release of damage-associated molecular patterns (DAMPs; such as mtDNA) and accumulation of succinate, thereby activating succinate receptor 1, enhancing immune inflammatory responses, and resulting in organ damage [86].

#### Heart failure

##### Mitochondrial morphology and dynamics

In heart failure, the expression of the mitochondrial fission-related proteins DRP1 and FIS1 is upregulated, while that of mitochondrial fusion-related proteins MFN2 and OPA1 and biogenesis-related protein PGC-1α is impaired. Additionally, the expression of the autophagy-related protein PINK1 decreased [24]. These changes result in fragmentation of myocardial mitochondria, disruption of inner cristae, vacuolar degeneration, and a decrease in mitochondrial size [87].

##### Mitochondrial function and metabolism

During heart failure, OXPHOS in the heart is severely impaired, mainly manifested as decreased activity of complexes I – IV. Mitochondrial dysfunction during heart failure also involves reduced production of cardiolipin and coenzyme Q (CoQ) [24], heightened generation of ROS, disturbances in calcium metabolism, excessive opening of mPTP, and depletion of ATP [88]. In terms of mitochondrial metabolism, fatty acids are the main sources of ATP in a healthy heart; however, during heart failure, the expression and activity of the fatty acid conversion enzymes carnitine palmitoyltransferase 1 and 2 decreased, leading to impaired fatty acid metabolism. Additionally, the expression of glucose transporter 1 increased, causing a shift in the energy substrate preference from mitochondrial fatty acid oxidation to glucose metabolism [24]. Furthermore, glycolysis and ketone oxidation increased, while amino acid oxidation and lactate oxidation decreased [89], indicating significant changes in the metabolic function of myocardial mitochondria during heart failure.

##### Mitochondrial protein expression and regulation

The expression of the deacetylase enzyme sirtuin 3 is downregulated in myocardial mitochondria after heart failure, leading to increased acetylation levels of complex I, aldehyde dehydrogenase 2, mitochondrial ribosomal protein L10, superoxide dismutase 2 (SOD2), long-chain acyl-CoA dehydrogenase, TFAM, PGC-1α, CYPD, leucine-rich protein 130 kD (LRP130), and LONP1. This dysregulation affects mitochondrial gene transcription and expression. SOD2 acetylation exacerbates oxidative stress damage in myocardial mitochondria. Acetylation of long-chain acyl-CoA dehydrogenase causes abnormal cardiac fatty acid oxidation, accelerating myocardial remodeling and the progression of heart failure. Additionally, CYPD acetylation increases the opening degree of mPTP channels, thereby aggravating myocardial cell apoptosis. Moreover, acetylation of LRP130 inhibits the transcriptional expression of mtDNA-encoded genes and reduces OXPHOS efficiency in myocardial cells [90]. Deleting *LONP1* in mouse cardiomyocytes impairs the integrity of mitochondria-endoplasmic reticulum contacts and mitochondrial fusion, leading to the activation of mtUPR within the mitochondria. This, in turn, results in heart-specific metabolic reprogramming and pathological cardiac remodeling [91].

#### Myocardial ischemia–reperfusion

##### Mitochondrial morphology and dynamics

Myocardial ischemia–reperfusion is characterized by excessive mitochondrial fission and reduced fusion [25]. The increased expression of the fission-related proteins DRP1, FIS1, and mitochondrial dynamics protein of 49/51 kD, as well as decreased expression of the fusion-related proteins MFN1 and OPA1 lead to mitochondrial fragmentation. Additionally, decreased FUN14 domain containing 1 (FUNDC1) phosphorylation hinders mitochondrial autophagy, further exacerbating ischemia–reperfusion injury [26, 27].

##### Mitochondrial function and metabolism

During the process of myocardial ischemia–reperfusion, the excessive opening of the mPTP channel led to an increased release of cytochrome C (Cyt C) and an upregulation of proteins like B-cell lymphoma 2 (BCL2) associated X apoptosis regulator and caspase-3/-9, accelerating the apoptosis of cardiomyocyte [27]. The ROS-mediated oxidation of cardiolipin and the dissociation of the channel structural proteins hexokinase 2 and voltage dependent anion channel 1 are potential mechanisms underlying excessive mPTP channel opening during myocardial ischemia–reperfusion [92].

##### Mitochondrial protein expression and regulation

Mitochondrial proteins underwent various modifications during myocardial ischemia–reperfusion, including phosphorylation, ubiquitination, and acetylation [93]. SUMOylation of DRP1 accelerates mitochondrial fission [94], and ubiquitination of mitochondrial Rho GTPase 2 accelerates its degradation, leading to impaired mitochondrial communication [95]. Furthermore, mtDNA mutations and changes in copy number hinder mitochondrial protein synthesis, exacerbating cardiac injury [96]. As an upstream regulatory factor, the FUN14 domain of FUNDC1 reduced mtDNA generation, thereby activating mtUPR. This leads to increased transcription levels of LONP1, CLPXP, and HSP10 to maintain mitochondrial quality control [97].

#### PAH

##### Mitochondrial morphology and dynamics

In the pulmonary arterial smooth muscle cells of PAH patients, increased expression of DRP1 and FIS1 and decreased expression of MFN2 lead to excessive mitochondrial fission and reduced fusion [28–30]. The downregulation of factors such as PGC-1α and TFAM results in a reduction in mitochondrial biogenesis [31]. FUNDC1 activates the ROS-hypoxia inducible factor 1 subunit α pathway, promoting mitochondrial autophagy and subsequently leading to sustained proliferation of pulmonary arterial smooth muscle cells [32].

##### Mitochondrial function and metabolism

Disruption of calcium regulation in the mitochondria is a major characteristic of PAH. In the smooth muscle cells of the pulmonary arterial, reduced levels of MCU and uncoupling protein 2 on the mitochondrial membrane hinder calcium transfer from the endoplasmic reticulum to mitochondria. This suppression of mitochondrial metabolic enzyme activity, such as pyruvate dehydrogenase [98], leads to a shift from aerobic oxidation to glycolysis as the main form of mitochondrial metabolism. Additionally, it inhibits apoptosis of pulmonary arterial smooth muscle cells [99, 100].

##### Mitochondrial protein expression and regulation

In PAH, the increased expression of the mitochondrial import inner membrane translocase subunits 13 and 9 proteins facilitated the import of folded proteins into the mitochondria, while the decreased expression of LONP1 diminished the ability of mitochondria to degrade misfolded proteins, leading to their accumulation [101]. Additionally, the downregulation of sirtuin 1 in PAH increased the acetylation of PGC-1α and forkhead box O1, as well as reduced protein expression, accelerating metabolic reprogramming [102].

### Manifestations of mitochondrial quality imbalance in inflammatory disorders

Inflammatory disorders are characterized by damage to tissues and organs due to the infiltration of inflammatory cells and the release of inflammatory mediators. Given the critical role of mitochondria in maintaining cellular homeostasis and controlling immune responses, the impact of inflammatory conditions on mitochondrial quality is significant. Examples of common inflammatory diseases are sepsis, acute pancreatitis, and bacterial meningitis.

#### Sepsis

##### Mitochondrial morphology and dynamics

During sepsis, there is an increase in mitochondrial fission and a decrease in fusion, leading to significant mitochondrial fragmentation. This is closely associated with the activation and increase of DRP1 and a decrease in the expression of MFN1 and MFN2 [33]. In the later stages of sepsis, downregulation of PGC-1α results in reduced mitochondrial biogenesis; moreover, decreased expression of PINK1, PARKIN, p62, and other factors impaired mitophagy, preventing the clearance of damaged mitochondria [33].

##### Mitochondrial function and metabolism

Sepsis also leads to increased production of ROS, reduced ATP generation, significantly decreased OXPHOS and MMP, elevated levels of lipid peroxidation, depletion of SOD2, severe mitochondrial calcium overload, and excessive opening of the mPTP channel in cardiac myocytes and vascular smooth muscle cells. These disruptions in mitochondrial function and metabolism further contribute to increased apoptosis, exacerbating the progression of the disease [33, 103, 104].

##### Mitochondrial protein expression and regulation

In sepsis, cells in a state of death or imminent death release DAMPs like mtDNA, which activate innate immune cells through the cyclic guanosine monophosphate-adenosine monophosphate (GMP-AMP) synthase-stimulator of interferon gene signaling pathway, leading to the release of cytokines, such as tumor necrosis factor-α (TNF-α) and interleukin (IL)-1β [105]. Notably, mtDNA can also serve as a metabolic-associated molecular marker that accelerates the progression of sepsis through the Toll-like receptor 9 (TLR9)-NLR family pyrin domain containing 3 signaling pathways. In neutrophils, binding of mtDNA to TLR9 promotes the release of matrix metalloproteinases-8 and -9 along with other proinflammatory factors [106]. Additionally, lipopolysaccharide-induced sepsis leads to mild upregulation of mtUPR in cardiomyocytes, and inhibiting mtUPR compromises the protective benefits of FUNDC1-mediated mitochondrial autophagy in cardiomyocytes [107].

#### Acute pancreatitis

##### Mitochondrial morphology and dynamics

In acute pancreatitis, pancreatic cells exhibit swollen mitochondria, disruption of the mitochondrial matrix and loss of mitochondrial cristae. These changes are primarily associated with increased FIS1 expression and decreased expression of OPA1 and MFN2 [34]. The heightened expression of PARKIN and accumulation of p62 trigger mitochondrial autophagy in pancreatic cells [34, 35]. Additionally, in peritoneal macrophages, the elevated expression of PGC-1α promotes mitochondrial biogenesis, contributing to the development of pancreatitis [36].

##### Mitochondrial function and metabolism

Various factors that cause acute pancreatitis result in harm to acinar and ductal cells by impairing normal intracellular calcium signaling [108]. This damage leads to the release of calcium from the endoplasmic reticulum, an overload of calcium in the mitochondria, and subsequent excessive opening of the mPTP channel, resulting in MMP reduction and mitochondrial dysfunction [109]. The dysfunction hinders the maintenance of endoplasmic reticulum and plasma membrane calcium ATPase pumps, further worsening calcium toxicity and ultimately leading to cell death [34, 110].

##### Mitochondrial protein expression and regulation

During pancreatitis, damaged or necrotic cells release DAMPs, including mtDNA and high mobility group box 1. These DAMPs, through TLR signaling pathway, promote the formation and release of inflammasomes, ultimately activating inflammatory and immune stress responses [35].

#### Bacterial meningitis

##### Mitochondrial function and metabolism

In streptococcal meningitis, the lactate/pyruvate ratio in the brain is elevated. This change leads to mitochondrial metabolic dysfunction, resulting in imbalanced energy metabolism within the brain [111]. Pneumococcal meningitis is associated with pneumolysin causing an increase in intracellular Ca^2+^ levels, which trigger the release of apoptosis-inducing factors from the mitochondria and cell apoptosis [112]. During *Neisseria meningitidis* infection, the bacterium binds to mitochondria in target cells through its porins, stabilizing MMP, reducing the release of Cyt C, and promoting viral dissemination [113].

##### Mitochondrial protein expression and regulation

In the presence of *Streptococcus suis* serovar 2 infection, a pathogenic factor called enolase induces HSP family D member 1 translocation from mitochondria to the cytoplasm. After translocation, ytoplasmic HSP family D member 1 can interact with β-actin, leading to mitochondrial apoptosis [114].

### Manifestations of mitochondrial quality imbalance in viral infections

Viral infections like viral hepatitis, human immunodeficiency virus (HIV), and coronavirus disease 2019 (COVID-19), are caused by the invasion and replication of viruses, leading to tissue or organ damage. The entry and replication of viruses in cells directly affect the quality of cellular mitochondria, disrupting cellular energy metabolism and exacerbating disease progression. The lifecycle of viruses is also closely related to mitochondrial quality.

#### Viral hepatitis

##### Mitochondrial morphology and dynamics

In hepatitis B virus (HBV) infection, the HBV X protein (HBX) has various effects on mitochondrial morphology and dynamics [37]. This protein enhanced DRP1 function and translocation, leading to excessive mitochondrial fission and increased mitophagy [38]. It also reduced the movement of mitochondria along microtubules, resulting in the perinuclear accumulation of fragmented mitochondria [39]. Additionally, HBX induces the expression of PINK1 on the outer mitochondrial membrane and recruits a large amount of PARKIN to promote the degradation of damaged mitochondria, preventing cell apoptosis and leading to the long-term survival of HBV-infected cells and viral persistence [38, 40]. In patients with hepatitis C virus (HCV), alterations in hepatocyte mitochondria are also common [41]. HCV infection enhances the production of mitochondrial autophagosomes, but the HCV core protein inhibits the recruitment of PARKIN by damaged mitochondria, thereby obstructing the fusion of mitochondrial autophagosomes with lysosomes and impeding the degradation of damaged mitochondria [42], which in turn promotes viral replication.

##### Mitochondrial function and metabolism

Owing to its crucial metabolic functions, the liver contains approximately 1000 – 2000 mitochondria per hepatocyte. Viral hepatitis is often accompanied by severe mitochondrial dysfunction and metabolic disorders [115]. The HBX causes a loss of MMP, disrupts OXPHOS, inhibits ATP production, and induces the release of Cyt C by interacting with p53 or voltage dependent anion channel 3 on the outer mitochondrial membrane [116]. It also interacts with complex III, leading to increased production of ROS [117] and release of mitochondrial calcium [37]. The HCV-encoded core protein induces ROS production and impairs mitochondrial function by inhibiting the ETC in mitochondria [118]. Furthermore, both HBV and HCV can affect the membrane channels between mitochondria and the endoplasmic reticulum, resulting in functional impairment of mitochondrial calcium transport [116, 119].

##### Mitochondrial protein expression and regulation

After being infected with HBV and HCV, the expression of normal mitochondrial proteins in hepatocytes is severely disrupted. These viruses localize and replicate within mitochondria, then trigger chronic inflammation and continuous production of viral antigens through signal transduction pathways, depleting the mitochondrial function of the specific T cells targeting them [120, 121]. HBX promoted the mtUPR and increased LONP1 expression [40], stimulating the release of damaged mtDNA and further promoting the occurrence and development of hepatitis through the TLR signaling pathway [37]. In host defense regulation, the interaction between HBX and MAVS downregulates antiviral immune responses while upregulating pro-inflammatory mediators, such as interferons and interleukins. HCV also suppresses cell apoptosis through the MAVS signaling pathway, contributing to viral infection persistence [122]. The activity of the mtUPR in the livers of HBV and HCV patients undergoes significant alterations. The expression of LONP1 was upregulated in HBV patients, whereas the expression of HSP60 and HSP70 were significantly downregulated. These changes lead to the degradation of misfolded proteins, thereby promoting the survival and replication of the virus [123].

#### HIV

##### Mitochondrial morphology and dynamics

Following HIV infection, the envelope protein (ENV) of the virus upregulated MFN1 expression and inhibited DRP1 expression, thereby interfering with mitochondrial morphology dynamics [43]. The transactivator of transcription (TAT) protein of HIV induced high expression and phosphorylation of DRP1, leading to a change in mitochondrial shape from a reticular structure to a fragmented form [44]. TAT also inhibited mitochondrial autophagy flux by increasing p62 expression, resulting in impaired degradation of damaged mitochondria [45]. In cellular models, overexpression of the viral protein R of HIV leads to a reduction in MFN2 expression, decreased fusion of mitochondria, and fragmentation of mitochondria with altered cristae structure [46].

##### Mitochondrial function and metabolism

HIV infection causes T lymphocytes to exhibit high sensitivity to the loss of MMP, making them prone to mitochondrial-mediated apoptosis [124], leading to the depletion of immune cells, such as CD4^+^ T cells, macrophages, and monocytes [125, 126]. The depletion of T cells during HIV infection is closely related to mitochondrial metabolic dysfunction [127]. T cells showed increased glucose metabolism and glycolysis, which are markers of both inflammatory cells and essential metabolic pathways to produce certain cytokines [128, 129]. HIV infection also results in the reprogramming of immune cell metabolism, upregulating almost all metabolic branches, including glucose, lipid, and tryptophan metabolism [130].

##### Mitochondrial protein expression and regulation

The activity of HIV is strongly associated with the nuclear-encoded mitochondrial protein NLR family member X1 (NLRX1) and FAST kinase domains 5. In CD4^+^ T cells, the highest level of HIV is positively correlated with the expression of NLRX1. NLRX1 interacts directly and specifically with FAST kinase domains 5, thereby increasing the expression of components of ETC complexes I – V [131].

#### COVID-19

##### Mitochondrial morphology and dynamics

In COVID-19, nonstructural protein 7/9 of the severe acute respiratory syndrome coronavirus 2 (SARS-CoV-2) promotes the phosphorylation of DRP1 in lung epithelial cells, increasing the expression levels of DRP1 and FIS1 and facilitating mitochondrial fission [47, 48]. SARS-CoV-2 also upregulates the expression of the mitochondrial deubiquitinase ubiquitin-specific peptidase 30, inhibiting MFN1 and MFN2 expression and reducing mitochondrial fusion [48]. Furthermore, SARS-CoV-2 activates the host cell PINK1/PARKIN pathway to initiate mitochondrial autophagy while simultaneously inhibiting the binding of p62 to LC3, thereby impeding p62-labeled mitochondria engulfment by lysosomes and resulting in blocked mitochondrial autophagy flux [49]. The regulation of mitochondrial morphology and dynamics by SARS-CoV-2 plays a crucial role in viral replication and immune evasion.

##### Mitochondrial function and metabolism

The nonstructural protein 10 of SARS-CoV-2 specifically interacts with mitochondrially encoded NADH: ubiquinone oxidoreductase core subunit 4L (a subunit of respiratory chain complex I) and cytochrome oxidase II (the second subunit of respiratory chain complex IV), thereby disrupting the function of complex I in the ETC and leading to impaired OXPHOS characterized by a reduced rate of oxygen consumption and decreased ATP production [47, 132]. Additionally, viral RNA can enter mitochondria through translocase of outer mitochondrial membrane (TOM)20, causing loss of MMP, the opening of the mPTP channel, and accumulation of ROS [49]. In lung tissue samples from COVID-19 patients, activation of the ROS-hypoxia-inducible factor-1α pathway in alveolar macrophages exacerbated pro-inflammatory reactions [133]. Moreover, the activation of the interferon α-inducible protein 27 pathway disrupted immune cell mitochondrial energy metabolism, leading to a cytokine storm. Furthermore, disruption of the MCU channel in lung epithelial cells exacerbates viral immune evasion and the viral replication process [133].

##### Mitochondrial protein expression and regulation

The nonstructural protein 1 of SARS-CoV-2 inhibits the translation of the mitochondrial proteins TOM22 and TOM40, thereby suppressing the degradation of viral and host misfolded proteins [134]. Furthermore, coronaviruses can localize to mitochondria and interfere with the interaction between mitochondrial protein TOM70 and MAVS through signaling pathways, thereby inhibiting antiviral cellular responses and sustaining viral replication [135].

### Manifestations of mitochondrial quality imbalance in metabolic dysregulations

Metabolic dysregulations refer to the dysfunction of tissues and organs due to disruptions in metabolic processes. Mitochondria, which are responsible for cellular energy metabolism, are significantly affected by metabolic dysregulations. Common metabolic diseases include diabetes, obesity, and hyperlipidemia.

#### Diabetes

##### Mitochondrial morphology and dynamics

Patients with type 2 diabetes often exhibit decreased expression of the autophagy-related proteins PINK1 and PARKIN [50], increased expression of the fission-related protein DRP1, and decreased expression of the fusion-related proteins MFN2 and OPA1 [51, 52]. This leads to excessive mitochondrial fission and impaired clearance of damaged mitochondria. In the skeletal muscles of type 2 diabetes patients, decreased expression of PGC-1α is accompanied by reduced mitochondrial biogenesis, which is an important mechanism underlying the development of this disease [53].

##### Mitochondrial function and metabolism

The islets derived from patients with type 2 diabetes showed impaired calcium signaling and insulin secretion in response to glucose stimulation. Additionally, their skeletal muscles exhibited a reduction in the activity of mitochondrial oxidative enzyme and lipid oxidation, leading to the progression of disease [136]. High glucose levels can induce damage to mesangial and renal tubular epithelial cells as well as complexes I and III, ATP depletion, increased release of mitochondrial reactive oxygen species (mtROS), followed by lipid peroxidation and glycation modification of related proteins, and increased release of pro-apoptotic factors, such as Cyt C and apoptosis-inducing factor [137]. Moreover, mitochondrial dysfunction affects insulin signaling, synthesis, and secretion pathways, leading to insulin sensitivity/resistance in peripheral muscle tissue [138, 139]. Adipose tissue in patients with type 2 diabetes often showed increased production of pro-inflammatory factors, such as TNF-α, monocyte chemoattractant protein-1, and IL-6, which promote chronic inflammation of the pancreas and apoptosis of pancreatic β cells. This in turn contributed to the development of insulin resistance and induced sustained low-grade inflammation in patients with diabetes [140].

##### Mitochondrial protein expression and regulation

Currently, more than 28 mtDNA mutation sites have been identified in type 2 diabetes patients, including 4 mutation sites in the D-loop region (m.T16189C, m.T16519C, m.T58C, and m.151C) [140]. These mutations affect the copy number of mtDNA and overall mitochondrial function, leading to a reduction in ATP production, promotion of ROS generation, and damage to pancreatic β cells. This subsequently affects insulin secretion and worsens disease progression [140]. In the early stages of diabetes, activating transcription factor 5 (ATF5) can exert a protective effect through the mtUPR. However, in the presence of diabetic nephropathy, ATF5 in renal tissues can promote tubulointerstitial injury by regulating the expression levels of HSP60 and LONP1 [141].

#### Obesity

##### Mitochondrial morphology and dynamics

The propensity for liver fat accumulation is heightened in obese individuals, thereby predisposing them to the development of non-alcoholic fatty liver disease (NAFLD). The expression of DRP1 and mitochondrial fission factor was upregulated in NAFLD-affected hepatocytes, while the expression of MFN1 and MFN2 was downregulated. Reduced activity of the PINK1/PARKIN and BCL2 interacting protein 3-like pathways results in impaired mitochondrial autophagy [54]. Hepatic mitochondria exhibit swelling, loss of cristae, and the presence of megamitochondria with paracrystalline inclusion bodies [55]. In adipose tissue, decreased expression of PGC-1αleads to reduced mitochondrial biogenesis, inhibiting the conversion of free fatty acids and triglycerides into adipose tissue [56].

##### Mitochondrial function and metabolism

In NAFLD, the excessive influx of free fatty acids into the liver impairs β-oxidation, resulting in the accumulation of lipotoxic substances, inflammation, and disruption of insulin signaling [55]. The excess NADH and flavin adenine dinucleotide hydrogen generated by mitochondrial β-oxidation transfer excess electrons to the ETC, disrupting electron flow within the ETC. This caused electron leakage and ROS production, triggering hepatocyte stress, and driving the progression of NAFLD [55]. Increased ROS also damaged lipids, proteins, and DNA, induced apoptosis signaling, and affected peripheral tissues, the blood–brain barrier, and the central nervous system [142]. In obese individuals, reduced activity of OXPHOS complexes I – IV in subcutaneous adipose tissue led to decreased utilization of mitochondrial phosphate and loss of MMP, resulting in abnormal fat conversion in adipocytes [143]. After obesity onset, reduced expression of glucose transporter type 4 on the adipocyte membrane decreased glucose uptake by adipose tissue and subsequently limited acetyl-CoA availability for the TCA cycle [56].

##### Mitochondrial protein expression and regulation

In obese adipocytes, there is a reduction in mtDNA content, as well as decreased levels of mitochondrial ribosomal and OXPHOS transcripts, and subunit protein, which ultimately leads to impaired adipocyte function [56]. Similarly, patients with NAFLD exhibit reduced mtDNA content in hepatocytes, with an increased frequency of mtDNA mutations, compared with that in healthy individuals. The severity of liver fibrosis is associated with an increased number of mtDNA mutation sites in the liver [144]. These mutated genes are mainly involved in the expression of OXPHOS proteins [144]. In both mouse and human models of NAFLD, activation of the mtUPR leads to increased expression of HSP60 and HSP90, resulting in the accumulation of lipids in the liver and promoting hepatic injury [145].

#### Hyperlipidemia

##### Mitochondrial morphology and dynamics

In hyperlipidemia, DRP1 expression increased, while MFN1 expression decreased in cardiac tissue, resulting in excessive fission and fragmentation of mitochondria. However, there were no significant changes in OPA1 and MFN2 expression [57]. In muscle and liver tissues, the expression of PGC-1α, MFN1, MFN2, and OPA1 was reduced, thereby decreasing mitochondrial biogenesis and fusion. However, FIS1 expression remains relatively unchanged [58]. In endothelial cells, the expression of PARKIN and BNIP3 is reduced, impairing mitochondrial autophagy [59]. In subcutaneous and visceral adipose tissues, there were no significant changes in the expression of proteins, such as PINK1, PARKIN, and FUNDC1, nor mitochondrial autophagy apparent [60].

##### Mitochondrial function and metabolism

In individuals with high levels of fats in their blood, known as hyperlipidemia, elevated levels of free fatty acids can inhibit structural proteins (such as adenosine nucleotide translocase and UCPs) of the mPTP channel. This can lead to excessive opening of the mPTP channel, decreased MMP, reduced ATP production, and extensive accumulation of lipid droplets. These effects exacerbate insulin resistance, inflammation, and cellular toxicity, ultimately contributing to the development of cardiovascular diseases, such as atherosclerosis [146]. Disruptions in the metabolism of myocardial lipids can result in an imbalance between the oxidation of fatty acid and the activity of ETC in mitochondria [147]. Increased ROS in cardiac myocytes induces various effects, including the modulation of key factors involved in myocardial energy substrate metabolism, such as AMP-activated protein kinase and peroxisome proliferator-activated receptor α/γ, disrupting insulin signaling, regulation of calcium channels, and oxidative-reductive modifications of transporters, such as ryanodine receptor 2 and sarcoendoplasmic reticulum calcium ATPase 2, leading to structural disturbances in cardiac myocytes and changes in cardiac function [147]. In cardiac tissue affected by hyperlipidemia, increased expression of BCL2-associated X apoptosis regulator activates pro-apoptotic signaling, increasing the myocardial infarction area [57].

### Manifestations of mitochondrial quality imbalance in degenerative conditions

Degenerative conditions are characterized by the dysfunction of tissues and organs caused by cellular aging or functional deterioration. The effect of degenerative conditions on mitochondrial quality is important because mitochondria play a central role in the life cycle of cells. A decrease in mitochondrial function or quality can result in insufficient production of cellular energy, triggering or exacerbating the decline in cellular functional, ultimately contributing to the occurrence and progression of degenerative diseases. The common degenerative diseases encompass the process of aging as well as neurodegenerative disorders such as Alzheimer’s disease (AD) and Parkinson’s disease (PD).

#### Aging

##### Mitochondrial morphology and dynamics

The impact of mitochondrial morphology and dynamics on the lifespan of organisms has been a topic of discussion. Impairment of both fission and fusion proteins has been reported in aging conditions. Sharma et al. [61] revealed that aging organs experience excessive mitochondrial fission and increased mitochondrial autophagy, leading to fragmented mitochondria. Increasing mitochondrial fusion and biogenesis may have beneficial effects on lifespan extension; however, long-term driving of fusion through *DRP1* knockout does not extend the lifespan of *Caenorhabditis elegans* [62]. Rana et al. [63] found a decline in the number of mitochondrial division sites associated with aging, but excessively high division rates can shorten lifespan. Additionally, a study conducted by Hong et al. [64] showed that in the skeletal muscle cells of aged mice, DRP1 expression and DRP-Ser616 phosphorylation decreased, accompanied by impaired mitochondrial autophagy. However, Baker et al. [65] demonstrated that OPA1 expression is significantly decreased and MFN1 expression is increased in aging muscle cells, but the expression of MFN2, FIS1, and DRP1 remains unchanged, leading to reduced mitochondrial fusion and fragmentation and compromising cellular self-renewal and normal function maintenance. These studies indicate that the pattern of changes in mitochondrial morphology and dynamics in aging mitochondria are not yet fully understood. Currently, a single reduction in fission or increase in fusion alone cannot effectively slow the aging process. A multidimensional understanding of the aging process requires consideration of the dynamic network of mitochondria.

##### Mitochondrial function and metabolism

As individuals age, the accumulation of ROS leads to increased permeability of mitochondrial membrane, which in turn causes chronic inflammation and cell death [148]. Aging also leads to a decrease in both fatty acid oxidation and mitochondrial turnover decrease, resulting in reduced cellular adaptability and excessive activation of inflammasomes [149].

##### Mitochondrial protein expression and regulation

Plasma levels of mitochondrial microproteins, such as RNR2 (an mtDNA-encoded rRNA, human), gradually decrease with aging. The high expression levels of RNR2 in people who live to be 100 years suggest a potential association between RNR2 expression and lifespan extension [149]. Study has demonstrated that introducing RNR2 into nematodes can extend their lifespan by triggering autophagy [149]. Another mtDNA-encoded protein, RNR1 (an mtDNA-encoded rRNA, mitochondrial open reading frame of the 12S rRNA-c), also decreases gradually with aging and has been found to help prevent age-related insulin resistance and obesity [149]. Additionally, communication between mitochondria and the cell nucleus can stimulate signaling pathways by influencing mitochondrial metabolites or stress, leading to various epigenetic changes that affect the aging process [150]. Components of mtUPR, LONP1, HSP60, and HSP70 undergo significant changes during the aging process. HSP60, in particular, is closely associated with long lifespans in various animal models, such as the heart, livers, brain, and multiple organs [151]. Furthermore, the absence of the CLPXP protease can also promote the aging process [151].

#### AD

##### Mitochondrial morphology and dynamics

Previous studies have suggested a close association between pathological changes in AD and the deposition of amyloid-β (Aβ) and tau proteins. However, recent research has indicated that AD severity is not significantly correlated with Aβ deposition, suggesting that it may not be the key driving factor in AD [66]. Instead, widespread mitochondrial damage in the early stages of AD appears to be the primary cause of disease progression [67]. In a mouse model of AD, the expression of proteins involved in mitochondrial fission (DRP1 and FIS1) increased, while those involved in fusion (MFN1, MFN2, and OPA1) and factors related to biogenesis (PGC-1α, NRF1, NRF2, and TFAM) decreased [68]. Research has shown that reducing the expression of DRP1 in mice can decrease Aβ production and increase mitochondrial biogenesis and synaptic activity [69]. Additionally, elevated levels of Aβ and p-Tau can increase PINK1 phosphorylation and decrease mitochondrial PARKIN expression, leading to reduced mitochondrial autophagy [70].

##### Mitochondrial function and metabolism

In AD, mitochondrial metabolic capacity is significantly decreased, as evidenced by reduced enzyme activity in the TCA cycle and decreased ATP generation [152]. The presence of Aβ and tau proteins leads to a decrease in the expression of complex IV and impaired mitochondrial ETC enzyme activity, which in turn triggers neuronal dysfunction and metabolic reprogramming [153]. In AD patients, mutations in presenilin-1 and increased expression of alanine transaminase 2 results in reduced activity of complex I, increased interaction between the endoplasmic reticulum and mitochondria, accumulation of ROS, and impaired neuronal function [154]. Alterations in the distribution of mitochondrial membrane phospholipids and disruptions in calcium metabolism further exacerbate AD progression [152].

#### PD

##### Mitochondrial morphology and dynamics

PD is a condition that involves the degeneration of nerve cells that produce dopamine in a specific area of the brain. Reduced mitochondrial autophagy resulting from mutations in *PINK1* and *PARKIN*, which is an important mechanism underlying PD pathogenesis [71, 72]. Lower levels of PARKIN weaken the degradation process of ubiquitinated DRP1 [73]. Additionally, individuals with PD may experience reduced levels of sirtuin 1, which can lead to reduced activity of PGC-1α and decreased mitochondrial biogenesis [74].

##### Mitochondrial function and metabolism

Mitochondrial dysfunction plays a crucial role in the initial stages of dopaminergic neuron loss in PD. In PD patients, mutations in single genes like those in synuclein α and leucine-rich repeat kinase 2 frequently result in mitochondrial dysfunction, oxidative stress due to dopamine self-degradation, damage to mtDNA, abnormal metabolism of mitochondrial phospholipids, and disruption of mitochondrial calcium homeostasis [14, 155, 156]. Additionally, complex I activity is inhibited, further exacerbating the loss of dopaminergic cells [71].

##### Mitochondrial protein expression and regulation

In the process of mtUPR in PD patients, downregulation the expression of HtrA serine peptidase 2 can lead to the development of PD-like symptoms. Additionally, the expression of LONP1 and CLPXP in PD models decreases. LONP1 and CLPXP improve PD-related phenotypes, respectively, by degrading PINK1 and parkinson disease protein 7, which are involved in the pathological process of PD, and reducing the accumulation of α-synuclein variant A53T [151].

### Manifestations of mitochondrial quality imbalance in tumors

The pathological characteristics of tumors include uncontrolled proliferation and unlimited expansion of abnormal cells, resulting in the formation of tumor masses with abnormal tissue structure, cellular pleomorphism, increased nuclear division, and invasive capabilities. The imbalance in mitochondrial quality is a crucial factor in the development of cancer [157, 158], as altered mitochondrial energy metabolism is a common feature of cancer itself [159]. Furthermore, mitochondria play a critical role in tasks, such as biosynthesis, signal transduction, cell differentiation, apoptosis, and maintenance of cell cycle and growth control, all of which are closely associated with tumor occurrence and development [158, 160].

#### Mitochondrial morphology and dynamics

Increasing evidence suggests that tumor cells can gain advantages in growth and survival by altering mitochondrial morphology and dynamics [75]. Increased mitochondrial fission is a common phenomenon in various types of tumors, including glioblastomas, melanoma, ovarian, breast, lung, and thyroid cancer [75]. Additionally, increased mitochondrial fusion is directly linked to chemoresistance in tumor cells [75]. In colorectal cancer, increased expression of MCU leads to enhanced uptake of mitochondrial Ca^2+^, promoting mitochondrial biogenesis by inhibiting TFAM phosphorylation, ultimately accelerating the proliferation of colorectal cancer cells [76]. In hepatocellular carcinoma, stomatin-like 2 promotes metastasis by activating PINK1-mediated mitochondrial autophagy to eliminate damaged mitochondria [77].

#### Mitochondrial function and metabolism

Owing to mutations in oncogenes, tumor suppressor genes, and metabolic enzymes, multiple mitochondrial metabolic pathways, including OXPHOS, fatty acid metabolism, glutamine metabolism, and one-carbon metabolism, undergo significant changes in tumors. This forms the theoretical basis of the Warburg effect in tumor metabolism. Abnormal mitochondrial metabolism in tumor cells induces metabolic reprogramming, favoring the utilization of glycolysis for ATP production and biosynthesis to meet growth and proliferation demands [161].

#### Mitochondrial protein expression and regulation

Pan-cancer analysis of whole genomes has revealed there are mitochondrial somatic mutations, mtDNA nuclear transfer, structural variations, and copy number alterations in various cancers. The amount of mtDNA nuclear transfer in tumors positively correlates with the number of structural variations in the nuclear genome [162]. The mtUPR is activated in tumors to maintain mitochondrial integrity and support tumor growth. When mitochondria are not functioning properly, they produce mtROS, which damages mitochondrial proteins and induces stress due to protein toxicity. In response, ATF5 triggers the upregulation of mitochondrial chaperones HSP60, HSP10, and mitochondrial HSP70, facilitating the refolding of proteins into their correct conformation. LONP1 and CLPXP also process additional damaged proteins that were not handled by the HSPs. The mtUPR ensures the integrity of mitochondria despite persistent oxidative and protein toxicity stress in cancer [163].

## Therapeutic strategies for mitochondrial quality control

**Fig. 2:**
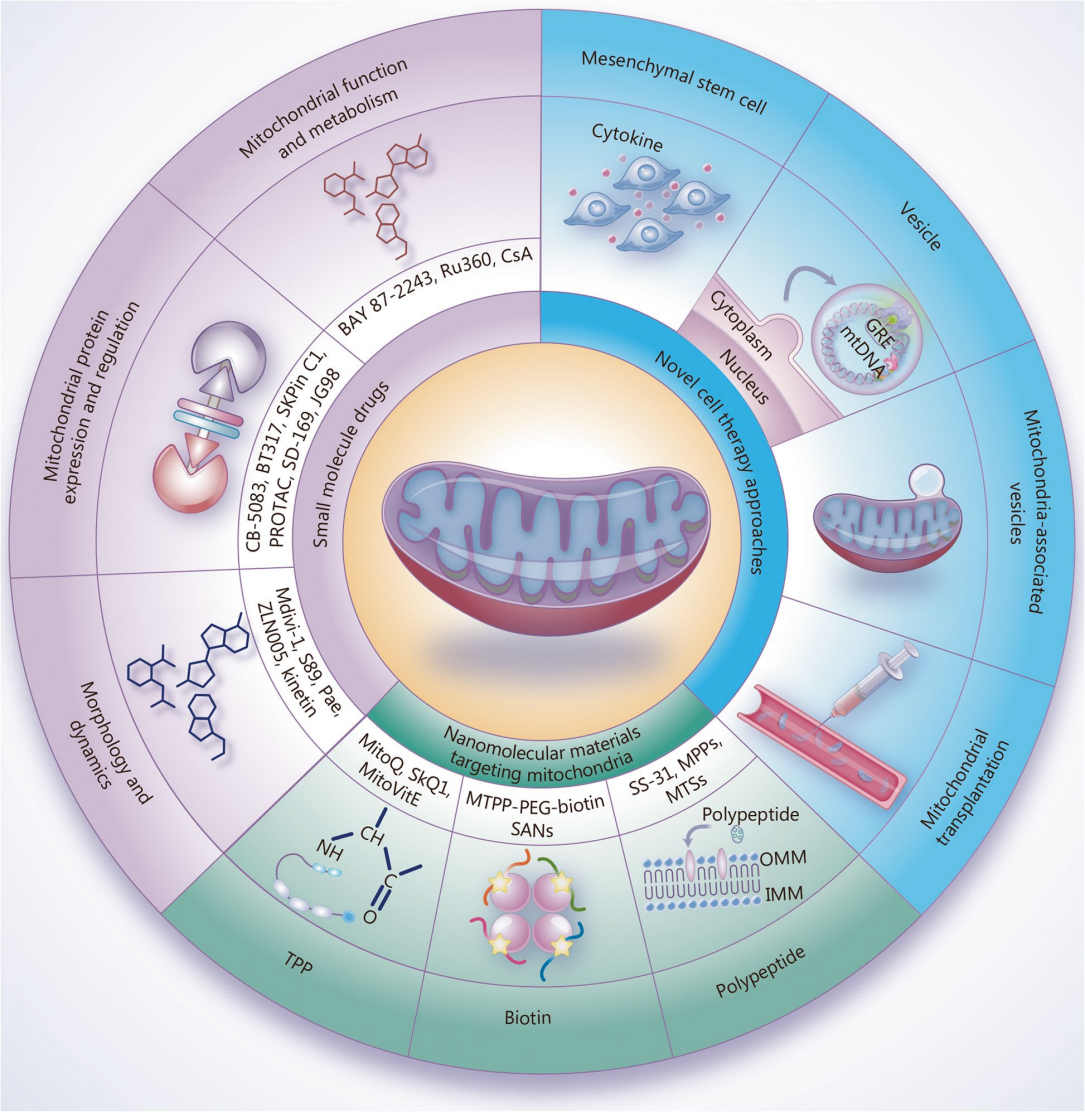
Therapeutic strategies targeting mitochondrial quality control. Effective interventions and treatment strategies targeting mitochondrial quality control in major diseases through small molecule drugs, nanomolecular materials, and novel cell therapy approaches. CsA cyclosporin A, BAY 87–2243 an inhibitor of hypoxia-induced gene activation, Ru360 an oxygen-bridged dinuclear ruthenium amine complex, GRE glucocorticoid response element (used here as an example for other gene regulatory elements), mtDNA mitochondrial DNA, CB-5083 an inhibitor of the p97 AAA ATPase, BT317 a dual LONP1 and chymotrypsin-like proteasome inhibitor, SKPin C1 a highly selective inhibitor of S-phase kinase associated protein 2, PROTAC proteolysis-targeting chimera, SD-169 a selective inhibitor of p38α MAPK, JG98 an allosteric modulator of heat shock protein 70, Mdivi-1 mitochondrial division inhibitor-1, S89 an agonist of mitofusin 1, Pae paeonol, ZLN005 an agonist of PGC-1α, TPP triphenylphosphonium, MTPP-PEG-biotin SANs biotin-conjugated pegylated photosensitizer self-assembled nanoparticles, SS szeto-schiller, MPPs mitochondrial penetrating peptides, MTSs mitochondrial targeting signal peptides, OMM outer mitochondrial membrane, IMM inner mitochondrial membrane

Nearly 400 different genes across two genomes of human have been associated with primary mitochondrial disease [164], and compromised mitochondria can directly impact cell survival and disease progression [165]. A comprehensive understanding of the specific manifestations of mitochondrial quality imbalance in major diseases is essential for developing effective interventions and treatment strategies. Currently, therapeutic strategies targeting mitochondrial quality control primarily focus on small molecule drugs that regulate key processes of mitochondrial quality, including morphology and dynamics, function and metabolism, protein expression and regulation, and novel approaches to mitochondrial therapy including nanomaterials and novel cellular therapies (Fig. 2).

**Fig. 3:**
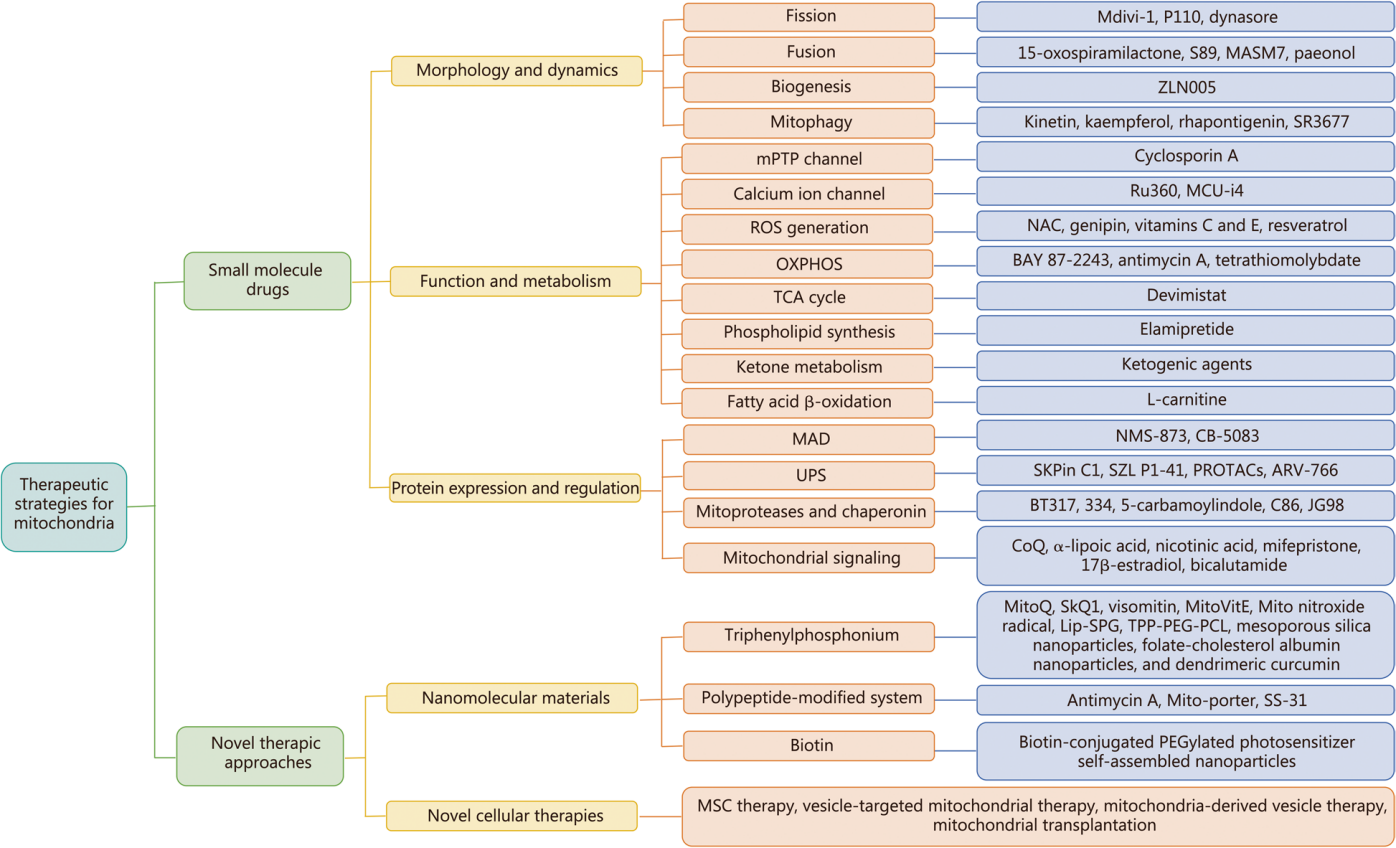
Mind mapping for therapeutic strategies targeting mitochondrial quality control. Therapeutic strategies aimed at enhancing mitochondrial quality control predominantly center on two key facets: small molecule drugs that modulate pivotal processes governing mitochondrial quality encompassing morphology and dynamics, function and metabolism, as well as protein expression and regulation; and innovative approaches to mitochondrial therapy, which encompass the utilization of nanomaterials and novel cellular therapies. ROS reactive oxygen species, mPTP mitochondrial permeability transition pore, OXPHOS oxidative phosphorylation, TCA tricarboxylic acid, MAD mitochondrial associated degradation, UPS ubiquitin–proteasome system, Mitoproteases mitochondrial protease system, MSC mesenchymal stem cell, Mdivi-1 mitochondrial division inhibitor-1, P110 a specific inhibitor of dynamin-related protein 1, S89 an agonist of mitofusin 1, MASM7 a serine protease activator, ZLN005 an agonist of PGC-1α, SR3677 Rho kinase inhibitor, Ru360 an oxygen-bridged dinuclear ruthenium amine complex, MCU-i4 mitochondrial calcium uniporter inhibitor, NAC N-acetyl-L-cysteine, BAY 87–2243 an inhibitor of hypoxia-induced gene activation, NMS-873 an allosteric inhibitor of p97, CB-5083 an inhibitor of the p97 AAA ATPase, SKPin C1 a highly selective inhibitor of S-phase kinase associated protein 2, SZL P1-41 specific inhibitor of S-phase kinase-associated protein 2, PROTACs proteolysis-targeting chimeras, ARV-766 luxdegalutamide, BT317 a dual LONP1 and chymotrypsin-like proteasome inhibitor, 334 an AAA ATPase inhibitor, C86 and JG98 allosteric modulators of heat shock protein 70, CoQ coenzyme Q, TPP-PEG-PCL triphenylphosphonium-poly(ethylene glycol)-poly(ε-caprolactone) polymers, Lip-SPG multi-targeted redox-sensitive liposomes, SS szeto-schiller

To facilitate understanding, we present the treatment strategy of mitochondria in the form of a mind map (Fig. 3) to more clearly target the treatment links of mitochondria. Additionally, we summarized small molecule drug therapy of mitochondria in Table 2 [14, 16, 17, 24, 165–253], and subsequently outlined new methods for mitochondrial therapy to enhance our understanding of the latest promising modes of targeted therapy.

**Table 2:** Small molecule drugs targeting mitochondrial quality control

Small molecule drug	Targeted protein	Targeting step	Research state	Reported disease models	References
Mdivi-1	DRP1	Fission	Preclinical phase	Cardiac arrest, heart failure, myocardial infarction, neurodegenerative diseases, acute kidney injury, and cerebral ischemia–reperfusion	[166–172]
P110	DRP1	Fission	Preclinical phase	Myocardial ischemia–reperfusion, sepsis-induced myocardial disease, PAH, sepsis-induced brain dysfunction, neurodegenerative diseases, and ALS	[25, 172–178]
Dynasore	DRP1	Fission	Preclinical phase	Spinal cord injury, myocardial ischemia–reperfusion, type 2 diabetes, and PD	[166, 179–182]
15-oxospiramilactone (S3)	MFN1/2	Fusion	Preclinical phase	Type 2 diabetes	[183, 184]
S89	MFN1	Fusion	Preclinical phase	Myocardial ischemia–reperfusion	[185]
MASM7	MFN2	Fusion	Preclinical phase	Spinal cord injury	[186, 187]
Paeonol (Pae)	OPA1	Fusion	Preclinical phase	Inflammation, pain-related diseases, oxidative damage, diabetic cardiomyopathy, cardiovascular diseases, PAH, AD, dentine hypersensitivity, and soft tissue injury	[188–192]
ZLN005	PGC-1α	Biogenesis	Preclinical phase	Diabetes, neuroprotection, retinal protection, renal ischemia–reperfusion, and high glucose-induced cardiomyopathy	[193–197]
Kinetin	PINK1	Mitophagy	Phase I clinical trial (NCT02274051)	Familial dysautonomia	[198]
			Phase IV clinical trial (NCT01898182)	Skin photoaging	
Kaempferol	PINK1	Mitophagy	Phase I clinical trial (NCT06060691)	Female arousal disorder and sexual dysfunction	[199]
			Preclinical phase	AD	
Rhapontigenin	PINK1	Mitophagy	Preclinical phase	AD	[199]
SR3677	PARKIN	Mitophagy	Preclinical phase	Paraquat poisoning	[200]
Cyclosporin A (CsA)	/	mPTP channel	Phase II clinical trial (NCT01650662)	Reperfused acute myocardial infarction	[201–205]
			Preclinical phase	Pulmonary ischemia–reperfusion, hemorrhagic shock, and solid organ transplantation	
Ru360	/	Calcium ion channel	Preclinical phase	Myocardial ischemia–reperfusion, traumatic brain injury	[206, 207]
MCU-i4	/	Calcium ion channel	Preclinical phase	Cancer, pancreatitis, and heart failure	[208]
N-acetyl-L-cysteine (NAC)	/	ROS generation	Approved for listing	Dietary supplement	[209–211]
			Preclinical phase	Drug-induced liver injury, COVID-19, and PD	
Genipin	/	ROS generation	Phase III clinical trial (NCT05755750)	Horses with tendon injuries	[212, 213]
			Preclinical phase	Breast cancer, gastric cancer, liver cancer, and other tumor models, and tendon injuries	
Vitamins C and E	/	ROS generation	Approved for listing	Dietary supplement	[214, 215]
			Preclinical phase	Peripheral nerve damage, dysmenorrhea, and pelvic pain	
Resveratrol	/	ROS generation	Approved for listing	Dietary supplement	[216, 217]
			Preclinical phase	Hypertension, diabetes, and tumor	
BAY 87–2243	/	OXPHOS	Phase I clinical trial (NCT01297530)	Advanced malignancies	[218, 219]
			Preclinical phase	Lung cancer and melanoma	
Antimycin A	/	OXPHOS	Preclinical phase	Cervical carcinoma	[220]
Tetrathiomolybdate	/	OXPHOS	Phase II clinical trial (NCT00195091)	Breast cancer	[221]
			Preclinical phase	Ovarian cancer, endometrial cancer, and non-small cell lung cancer	
CPI-613 (devimistat)	/	TCA cycle	Phase I/II clinical trial (NCT04593758)	Relapsed or refractory clear cell sarcoma of soft tissue	[222, 223]
			Phase II clinical trial (NCT05733000)	Advanced chemorefractory solid tumors	
			Phase II clinical trial (NCT05854966)	Relapsed or refractory acute myeloid leukemia (AML)	
			Phase III clinical trial (NCT03504410)		
			Phase II clinical trial (NCT04217317)	Refractory T-cell non-hodgkin lymphoma	
			Phase I/II clinical trial (NCT04203160)	Advanced unresectable biliary tract cancer (Bilt-04)	
Elamipretide	/	Phospholipid synthesis	Phase III clinical trial (NCT05162768)	Primary mitochondrial disease from nuclear DNA mutations (Npmd) (Nupower)	[224–226]
			Preclinical phase	Primary mitochondrial myopathy, traumatic brain injury	
Ketogenic agents	/	Ketone metabolism	—	Cardiovascular disease	[15]
L-carnitine	/	Fatty acid β-oxidation	Approved for listing	Dietary supplement	[227, 228]
			Preclinical phase	Cardiovascular diseases, hypertension, hyperlipidemia, type 2 diabetes, and obesity	
NMS-873	/	MAD	Preclinical phase	Ovarian cancer	[17, 18, 229]
CB-5083	/	MAD	Phase I clinical trial (NCT02243917)	Advanced solid tumors	[17, 18, 229]
			Preclinical phase	Lymphoid system malignancies	
SKPin C1	/	UPS	Preclinical phase	Melanoma and myeloma	[230, 231]
SZL P1-41	/	UPS	Preclinical phase	Renal cancer, leukemia, glioma, and lung tissue fibrosis	[232–235]
PROTACs	/	UPS	—	Cancer, diabetic cardiomyopathy, and chronic myeloid leukemia	[236–239]
ARV-766	/	UPS	Phase II clinical trial (NCT05067140)	Prostate cancer	[240]
BT317	/	Mitoproteases and chaperonin	Preclinical phase	Tumor	[241]
334	/	Mitoproteases and chaperonin	Preclinical phase	Leukemia	[242]
SD-169 (5-carbamoylindole)	/	Mitoproteases and chaperonin	Preclinical phase	Diabetes	[243]
C86 (chalcone)	/	Mitoproteases and chaperonin	Phase II clinical trial (NCT06063655)	Prostate cancer	[244]
JG98	/	Mitoproteases and chaperonin	Preclinical phase	Prostate cancer	[244]
CoQ	/	Mitochondrial signaling	Approved for listing	Dietary supplement	[245–247]
			Preclinical phase	Acute kidney, lung, and septic liver injuries	
α-lipoic acid	/	Mitochondrial signaling	Approved for listing	Dietary supplement	[248]
			Preclinical phase	Sepsis	
Nicotinic acid (vitamin B3)	/	Mitochondrial signaling	Approved for listing	Dietary supplement	[249]
			Preclinical phase	Sepsis-induced liver injury	
Mifepristone	/	Mitochondrial signaling	Approved for listing	Type 2 diabetes, Cushing’s syndrome, and adenomyosis	[250, 251]
17β-estradiol	/	Mitochondrial signaling	Approved for listing	Cerebral ischemia	[252]
Bicalutamide (Bic, ICI-176334)	/	Mitochondrial signaling	Approved for listing	Tumors, such as prostate cancer	[253, 254]

*AD* Alzheimer’s disease, *PD* Parkinson’s disease, *COVID-19* coronavirus disease 2019, *ROS* reactive oxygen species, *mPTP* mitochondrial permeability transition pore, *OXPHOS* oxidative phosphorylation, *TCA* tricarboxylic acid, *MAD* mitochondrial associated degradation, *UPS* ubiquitin–proteasome system, Mitoproteases mitochondrial protease system, *Mdivi-1* mitochondrial division inhibitor-1, *P110* a specific inhibitor of dynamin-related protein 1, *S89* an agonist of mitofusin 1, *MASM7* a serine protease activator, *ZLN005* an agonist of PGC-1α, *SR3677* Rho kinase inhibitor, *Ru360* an oxygen-bridged dinuclear ruthenium amine complex, *MCU-i4* mitochondrial calcium uniporter inhibitor, *BAY 87–2243* an inhibitor of hypoxia-induced gene activation, *NMS-873* an allosteric inhibitor of p97, *CB-5083* an inhibitor of the p97 AAA ATPase, *SKPin C1* a highly selective inhibitor of S-phase kinase associated protein 2, *SZL P1-41* specific inhibitor of S-phase kinase-associated protein 2, *PROTACs* proteolysis-targeting chimeras, *ARV-766* luxdegalutamide, *BT317* a dual LONP1 and chymotrypsin-like proteasome inhibitor, 334 an AAA ATPase inhibitor, C86 and JG98 are allosteric modulators of heat shock protein 70, *CoQ* coenzyme Q, *DRP1* dynamin-related protein 1, *PAH* pulmonary arterial hypertension, *ALS* amyotrophic lateral sclerosis, *MFN* mitofusin, *OPA1* optic atrophy protein 1, *PGC-1α* peroxisome proliferator-activated receptor gamma coactivator-1α, *PINK1* phosphatase and tensin homolog (PTEN)-induced kinase 1, *PARKIN* parkinson protein 2 E3 ubiquitin protein ligase. Dietary supplements have been approved by the U.S. Food and Drug Administration (FDA, https://www.fda.gov/)

### Small molecule drugs that regulate critical steps in maintaining mitochondrial quality

Due to their excellent tissue penetration, small molecule drugs are extensively utilized for targeting mitochondria in medical treatment. Here, we highlight the crucial roles of relevant small molecule drugs in the treatment of major diseases by providing insights on mitochondrial morphology and dynamics, function and metabolism, as well as protein expression and regulation (Table 2).

#### Drugs targeting mitochondrial morphology and dynamics

The targeting of mitochondrial morphology and dynamics represents a rapidly growing field in the search for innovative therapeutic approaches to manage and treat various diseases by modulating mitochondrial functions. These interventions are focused on four primary strategies, including influencing mitochondrial fission, enhancing mitochondrial fusion, stimulating mitochondrial biogenesis, and promoting mitophagy (Fig. 1). Each strategy targets specific proteins or pathways, highlighting the intricate relationship between mitochondrial dynamics and cellular health.

##### Mitochondrial fission

The compounds mitochondrial division inhibitor-1 (Mdivi-1), P110 (a specific inhibitor of dynamin-related protein 1), and dynasore show promising therapeutic potential by targeting the DRP1 protein. Mdivi-1 selectively inhibits the assembly and GTPase activity of DRP1, thereby preventing mitochondrial fission [166, 167]. P110 specifically inhibits the interaction between DRP1 and FIS1, and directly binds to DRP1, inhibiting its GTPase activity [173]. Dynasore shares a similar mechanism with Mdivi-1 in inhibiting DRP1’s GTPase activity but distinguishes itself through protective effects against oxidative stress-induced mitochondrial fission [172]. By modulating mitochondrial fission, these drugs aim to maintain cellular health and provide new approaches for disease management. Mitochondrial fission is crucial for various cellular processes, and its dysregulation is linked to numerous pathological conditions.

##### Mitochondrial fusion

The discovery of a series of compounds, including 15-oxospiramilactone (S3), S89, MASM7 (a serine protease activator), and paeonol (Pae), have been identified to regulate mitochondrial fusion by targeting MFN1/2 and OPA1 proteins, enhancing our understanding of mitochondrial function regulation and offering potential therapeutic strategies for diseases associated with mitochondrial dysfunction.

##### Mitochondrial biogenesis

ZLN005 (an agonist of PGC-1α) is a remarkable drug that upregulates PGC-1α transcription, a key regulator of mitochondrial biogenesis. By boosting the transcription activity of factors such as myocyte enhancer factor 2, ZLN005 demonstrates effectiveness in ameliorating conditions including insulin resistance and dyslipidemia in diabetic mice [193], as well as providing neuroprotective and retinal protective effects [194, 195]. It also shows potential in alleviating renal ischemia–reperfusion injury [196] and high glucose-induced cardiomyopathy [197], emphasizing the significance of mitochondrial biogenesis in cellular health and disease.

##### Mitophagy

The regulation of mitophagy, essential for the elimination of damaged mitochondria, has gained attention due to its therapeutic potential. Compounds such as kinetin, kaempferol, rhapontigenin, and SR3677 (Rho kinase inhibitor) target the PINK1/PARKIN pathway, thereby promoting mitophagy and demonstrating potential applications in treating diseases characterized by compromised mitochondrial quality control.

#### Drugs targeting mitochondrial function and metabolism

Due to the complexities of mitochondrial function and metabolism, targeted drugs have been extensively investigated across various aspects. Cyclosporin A (CsA), for instance, impedes the opening of the mPTP, thereby attenuating the release of Cyt C and hindering the activation of the mitochondrial apoptosis pathway, while suppressing the expression of apoptosis-associated proteins. Ru360 (an oxygen-bridged dinuclear ruthenium amine complex) serves to inhibit the MCU channel, thus mitigating mitochondrial calcium overload. Meanwhile, MCU-i4 (mitochondrial calcium uniporter inhibitor) functions as an inhibitor of the mitochondrial calcium uptake 1 subunit of the MCU complex, thereby reducing mitochondrial Ca^2+^ intake. N-acetyl-L-cysteine (NAC), genipin, vitamins C and E, and resveratrol are therapeutic compounds with promising potential for alleviating oxidative stress-related conditions including metabolic disorders, cancer, and inflammatory conditions. Moreover, by targeting key enzymes and complexes involved in OXPHOS and the TCA cycle, researchers aim to disrupt the energy metabolism of cancer cells in order to reduce their proliferation and promote apoptosis. Prominent compounds currently under investigation include BAY 87–2243 (an inhibitor of hypoxia-induced gene activation), antimycin A, tetrathiomolybdate, and CPI-613 (devimistat), each designed to target specific components of metabolic pathways and demonstrating promising results across various cancer types. It is essential to highlight the significance of compounds that modulate phospholipid synthesis and ketone metabolism in maintaining cellular energy balance and mitochondrial function. In this context, elamipretide and ketogenic agents emerge as noteworthy contenders, offering innovative approaches and potential therapeutic prospects. L-carnitine plays a pivotal role in transporting fatty acid chains into the mitochondrial matrix, facilitating fat breakdown and energy acquisition from stored lipid reserves, thereby ameliorating oxygen saturation and impeding leukotriene synthesis. These pharmacological agents provide an effective means to address distinct aspects of mitochondrial function and metabolic pathways, thus paving the way for further clinical investigation and application (Table 2).

#### Drugs targeting mitochondrial protein expression and regulation

The MAD, UPS, mitochondrial protease system (mitoproteases), and chaperonins function as robust checkpoints for mitochondrial proteins, ensuring the efficient functioning of mitochondria. Notably, NMS-873 (an allosteric inhibitor of p97) and CB-5083 (an inhibitor of the p97 AAA ATPase) are specific valosin-containing protein allosteric inhibitors that can induce mitochondrial-associated ferroptosis or apoptosis, leading to cancer cell death. Targeting the UPS represents a promising therapeutic approach for combating various diseases, particularly cancer. Illustrative of this progress are compounds such as SKPin C1 (a highly selective inhibitor of S-phase kinase associated protein 2), SZL P1-41 (pecific inhibitor of S-phase kinase-associated protein 2), and proteolysis-targeting chimeras (PROTACs), each demonstrating distinct mechanisms and promising clinical prospects within UPS-based therapies. Furthermore, the mitochondrial protease system and chaperonins play pivotal roles in cellular homeostasis by regulating protein quality and turnover. Leading the way in treatments targeting these systems are compounds including BT317 (a dual LONP1 and chymotrypsin-like proteasome inhibitor), 334 (an AAA ATPase inhibitor), SD-169 (a selective inhibitor of p38α MAPK), C86 (allosteric modulator of heat shock protein 70), and JG98 (allosteric modulator of heat shock protein 70) (Table 2). It is noteworthy that mitochondrial signaling emerges as another viable therapeutic target. Current strategies for developing small molecule drugs directed at mitochondrial signaling predominantly involve cofactor supplementation or modulation of hormone receptors.

### Nanomolecular materials designed for precise targeting of mitochondria

The design and construction of nanomedicines are crucial for achieving precise targeting of mitochondria in the treatment of major diseases [255]. Currently, nanomaterials that target mitochondria include triphenylphosphonium (TPP), peptide modifications, and biotin, among others. These materials exhibit excellent biocompatibility and low toxicity, enabling specific binding to mitochondria and playing an important role in treating major diseases such as tumors, autoimmune diseases, and cardiovascular disorders (Table 3 [255–284]).

**Table 3:** Nanomolecular materials targeting mitochondria

Drug	Targeting step	Research state	Reported disease models	Reference
MitoQ	Triphenylphosphonium (TPP)	Approved for listing	Dietary supplement	[256–261]
		Preclinical research phase	AD, PD, and ischemia–reperfusion injury after kidney transplantation	
SkQ1	TPP	Phase II clinical trial (NCT02121301)	Keratoconjunctivitis sicca	[262–266]
		Phase III clinical trial (NCT03764735)	Dry eye syndrome	
		Phase III clinical trial (NCT04206020)	Dry eye syndrome	
		Preclinical research phase	PD, AD, aging	
MitoVitE	TPP	Preclinical research phase	Oxidative stress damage in the heart, brain, muscles, liver, and kidneys	[267, 268]
Mito nitroxide radical (TEMPO)	TPP	Preclinical research phase	Liver injury and PD	[269–272]
Lip-SPG	TPP	Preclinical research phase	Glioma	[273, 274]
TPP-PEG-PCL	TPP	Preclinical research phase	Lung cancer	[275]
Mesoporous silica nanoparticles	TPP	Preclinical research phase	Tumor	[276]
Folate-cholesterol albumin nanoparticles	TPP	Preclinical research phase	Tumor	[277]
Dendrimeric curcumin	TPP	Preclinical research phase	Tumor	[278]
Antimycin A	Polypeptide-modified mitochondrial targeting system	Recruiting (NCT04500938)	End-stage heart failure	[279]
		Recruiting (NCT05798260)	COVID-19 acute respiratory distress syndrome	
		Recruiting (NCT06003855)	Peripheral artery disease	
MITO-porter	Polypeptide-modified mitochondrial targeting system	Phase III clinical trial (NCT00003596)	Anal cancer	[280]
		Recruiting (NCT04166318)	Anal cancer	
		Phase III clinical trial (NCT01004978)	Hepatocellular carcinoma	
		Phase II clinical trial (NCT04216290)	Bladder cancer	
		Phase III clinical trials (NCT02528188)	Chronic pain and osteoarthritis	
SS-31	Polypeptide-modified mitochondrial targeting system	Recruiting (NCT05194631)	Diaphragm injury and mechanical ventilation complication	[281]
		Phase I/II clinical trial (NCT05168774)	Friedreich ataxia	
		Preclinical research phase	Acute myocardial infarction, stroke, and acute kidney injury	
Biotin-conjugated PEGylated photosensitizer self-assembled nanoparticles	Biotin	Preclinical research phase	Neurodegenerative diseases	[282–285]

*AD* Alzheimer’s disease, *PD* Parkinson’s disease, *COVID-19* coronavirus disease 2019, *TPP-PEG-PCL* triphenylphosphonium-poly(ethylene glycol)-poly(ε-caprolactone) polymers, *Lip-SPG* multi-targeted redox-sensitive liposomes, *SS* szeto-schiller. Dietary supplements have been approved by the U.S. Food and Drug Administration (FDA, https://www.fda.gov/)

#### Mitochondrial targeting by TPP

The targeting ability of TPP towards mitochondria is achieved through its reliance on the potential gradient across both inner and outer mitochondrial membranes. It functions as a carrier for delivering drugs specifically to mitochondria, which enables precise administration of antioxidants as well as other therapeutic agents. This ultimately leads to an enhancement in drug efficacy while simultaneously reducing drug dosage requirements [256]. Several small-molecule drugs conjugated with TPP have already been developed [286]. For instance, MitoQ, is created from combining TPP with CoQ, and has demonstrated effective targeting capabilities towards mitochondria for antioxidant purposes [256, 257]. Studies indicate that it has improved cognitive function in AD models [258, 259], reduced ischemia–reperfusion injury in a kidney transplantation model, and enhanced post-transplantation outcomes [260]. However, it did not show significant benefits when used to treat patients suffering from PD [261]. On the other hand, SkQ1, is another compound formed by combining TPP with plastoquinone. It exhibits similar antioxidant properties compared to MitoQ but possesses a distinct molecular structure. In animal models representing PD and AD, SkQ1 has shown promising results such as reduction in excessive phosphorylation of Aβ and tau proteins, improvement in memory and learning abilities, as well as the delay of mitochondrial dysfunction and neurodegenerative changes [262–264]. Furthermore, in a mouse model with mtDNA mutations, SkQ1 improves mitochondrial respiration capacity, delays age-related pathologies, and extends lifespan [265]. Visomitin, a derivative drug based on SkQ1, has successfully completed phase II clinical trials (NCT02121301) [266] and is currently undergoing phase III clinical trials in the United States (NCT03764735 and NCT04206020). SkQ1 has been approved for listing. MitoVitE, a mitochondria-targeted form of vitamin E formed by the combination of TPP and α-tocopherol, acts as a lipophilic antioxidant [267]. MitoVitE demonstrates anti-lipid peroxidation effects and protects mitochondria and cells from oxidative stress damage in the hearts, brain, muscles, livers, and kidneys [268]. The mitochondria-targeted nitroxide radical TEMPO formed by combining TPP with TEMPO possesses strong antioxidant properties. Mito-TEMPO improves mitochondrial function in a rat model of liver injury [269], reduces ROS production and lipid peroxidation induced by Aβ in a PD model [270], as well as prevents SOD2 inhibition and mtDNA damage [271, 272].

In addition to directly delivering antioxidant drugs, TPP can combined with other small molecule materials, such as liposomes, nanomicelles, folate, and dendritic polymers. This provides a wider range of possibilities and treatment strategies for personalized drug therapy in major diseases. For example, Tamam et al. [273, 274] and Peng et al. [273, 274] developed multi-targeted liposomes called Lip-SPG that are designed to target the brain, tumors, and mitochondria by co-modifying TPP with glucose. Lip-SPG enhances the uptake of doxorubicin and the chemosensitizer lonidamine by mitochondria in tumor cells. This effectively reduces ATP production, leading to increased ROS generation and decreased MMP levels, ultimately inhibiting tumor cell proliferation while inducing tumor cell apoptosis, thus treating glioma. Xu et al. [275] synthesized triphenylphosphonium-poly(ethylene glycol)-poly(ε-caprolactone) polymers (TPP-PEG-PCL) by combining TPP with nanomicelles loaded with gambogic acid and achieving mitochondrial targeting while inducing a decrease in MMP levels and release of Cyt C, thereby promoting tumor cell apoptosis, suggesting its potential application in lung cancer treatment. Lopez et al. [276] prepared mesoporous silica nanoparticles with dual targeting by co-modifying TPP with folate. These nanoparticles specifically target tumor cells by binding to folate receptors on the cell membrane and then enter mitochondria under the influence of MMP. Battogtokh et al. [277] combined TPP with docetaxel and loaded it onto folate-cholesterol albumin nanoparticles, demonstrating significant efficacy in anticancer therapy. Finally, Kianamiri et al. [278] developed targeted dendrimeric curcumin by combining TPP with polyamidoamine dendrimers loaded with curcumin, which efficiently delivers curcumin to tumor cell mitochondria and arrests the cell cycle at the G2/M phase, offering advantages such as targeting specificity, safety, and stability.

#### Polypeptide-modified mitochondrial targeting system

Peptides exhibit excellent biocompatibility and low toxicity. Mitochondria-penetrating peptides are synthetically designed for targeted mitochondrial delivery. For instance, Mallick et al. [279] formulated antimycin A, a hydrophobic drug, encapsulated in Chol-FRFK/D nanoliposomes using the MPP sequence of phenylalanine-arginine-phenylalanine-lysine. This composition disrupts the MMP in tumor cells to achieve antitumor effects. Mitochondrial targeting signal peptides (MTSs) penetrate mitochondria by binding to the outer mitochondrial membrane. Battogtokh et al. [280] developed MITO-porter, a nanoliposome surface modified with MTSs that enhances specific targeting to mitochondria by binding to TOM/translocase of inner mitochondrial membrane complexes on the outer mitochondrial membrane. Szeto-schiller (SS) peptides are tripeptides containing tyrosine, phenylalanine, and a basic amino acid. Unlike TPP, SS peptides have a lower reliance on MMP and mitochondrial integrity and are non-saturable. The SS peptide-based drug SS-31 specifically accumulates in the inner mitochondrial membrane through interaction with cardiolipin. This drug reduces mitochondrial ROS production, inhibits mPTP channel opening, and demonstrates anti-inflammatory, antioxidant, and organ-protective effects in major diseases, such as acute myocardial infarction, stroke, and acute kidney injury [281].

#### Mitochondrial targeting by biotin

Biotin demonstrates a strong binding affinity for tumor cells, enabling accurate detection of mitochondrial SO_2_ in these cells [282]. Biotin-conjugated PEGylated photosensitizer self-assembled nanoparticles serve as nano-carriers for chemical drugs and photosensitizer, specifically targeting mitochondria to rapidly induce the loss of MMP, thereby triggering apoptosis in tumor cell [283]. Additionally, exogenous supplementation of biotin can upregulate MFN2 to promote mitochondrial fusion, improve mitochondrial respiration, and potentially offer a therapeutic approach for neurodegenerative diseases [284, 285].

### Novel cellular therapy approaches

Despite achieving significant clinical outcomes in cancer and certain immune disorders [287, 288], the application of cell therapy in the field of mitochondrial-related diseases is still advancing. In recent years, mesenchymal stem cell (MSC) therapy has demonstrated broad potential in the treatment of various mitochondria-related major diseases. Additionally, innovative cellular therapy approaches, such as vesicle-targeted mitochondrial therapy, mitochondria-derived vesicle (MDV) therapy, and mitochondrial transplantation, have played a crucial role in modulating mitochondrial quality for the prevention and treatment of diverse major diseases.

#### MSC therapy

MSCs possess robust capabilities for self-renewal, regeneration, proliferation, and differentiation [289]. Recent studies have shown that maintaining mitochondrial quality control is a critical target for MSC-mediated tissue repair [290–292].

In the management of acute kidney injury, MSCs stimulate mitochondrial biogenesis in renal tubular epithelial cells by enhancing PGC-1α expression, thereby maintaining mitochondrial integrity. MSCs also facilitate mitochondrial transfer by directly transferring healthy mitochondria to damaged cells, aiding in their repair. Furthermore, MSCs secrete growth factors, such as vascular endothelial growth factor and hepatocyte growth factor, which reduce the expression of apoptosis-related proteins in mitochondria and improve mitochondrial function [293]. In the treatment of traumatic brain injuries, MSCs inhibit the expression of caspase-3 and secrete neuroprotective factors like nerve growth factor and vascular endothelial growth factor, thus restoring mitochondrial function, enhancing cellular energy metabolism, and protecting the mitochondria of damaged neurons [294]. In amyotrophic lateral sclerosis therapy, MSCs can decrease superoxide dismutase 1 aggregation and restore mitochondrial protein function through the secretion of extracellular vesicles [295]. When addressing liver failure following liver resection with MSCs intervention can improve hepatic cell mitochondrial function and promote lipid metabolism by secreting antioxidants and anti-inflammatory factors, thereby reducing oxidative stress and inflammation [296].

#### Vesicle-targeted mitochondrial therapy

Vesicle-targeted mitochondrial therapy has emerged as an innovative cellular therapeutic strategy, utilizing vesicles surrounded by a single-layered lipid bilayer membrane that include exosomes (30 – 50 nm in diameter), microvesicles (100 – 1000 nm in diameter), and apoptotic bodies (1 – 5 μm in diameter). These vesicles play a pivotal role in intercellular communication and substance exchange by transporting mitochondrial components or entire mitochondria to recipient cells [297].

Research findings indicate that vesicles play a crucial role in promoting mitophagy to counteract neurodegenerative diseases such as AD, while also contributing to cardiac health by preserving mitochondrial homeostasis via interactions within the macrophage network [298, 299]. The selective encapsulation of mitochondrial proteins effectively inhibits the release of mitochondrial DAMPs, protecting cells from inflammatory damage [300]. MSC-derived vesicles demonstrate potential in stabilizing mtDNA, mitigating both mitochondrial damage and inflammation [301]. Furthermore, they facilitate the transfer of mitochondria along with their associated components for treating conditions such as acute lung injury [302, 303], renal ischemia–reperfusion injury [304, 305], and neurodegenerative diseases [306, 307]. This process is also observed in energy-stressed adipocytes [302, 303, 308]. In cancer therapy, vesicle-mediated mitochondrial reprogramming suppresses tumor growth by regulating the mitochondrial metabolism of tumor cells [309]. Mitochondria-rich vesicles have shown promising results in restoring energy metabolism to ischemic myocardium while improving cardiac function among patients suffering from heart diseases, including heart failure [310]. In conclusion, vesicle-targeted mitochondrial therapy presents significant clinical potential not only for maintaining mitochondrial quality but also for safeguarding cellular health across a wide range of applications.

#### MDV therapy

MDVs are small vesicles originating from mitochondria that engage in interactions with other organelles via membrane structures and play roles in various processes, including mitochondrial-endoplasmic reticulum interactions, endoplasmic reticulum stress, and mitochondrial quality control [311]. MDVs regulate mitochondrial stress by modulating mitochondrial respiration, ROS generation, and MMP; influence the presentation of mitochondrial antigens; and transport lipids and peroxides to peroxisomes, thereby supplying metabolic substrates or enzymes for peroxisome biogenesis and proliferation [312].

Despite the demonstrated efficacy of MDVs in the treatment of major diseases, such as cardiovascular diseases, neurodegenerative diseases [313], tumors, and autoimmune diseases [314], their application in disease diagnosis and treatment is still at an early stage and faces numerous challenges. These challenges include a lack of a comprehensive understanding of the biological nature and functions of MDVs, inefficient and nonspecific methods for MDV isolation and identification, as well as uncertainties regarding the stability and biological distribution of MDVs in vivo and in vitro. Further research on MDVs will yield new insights and strategies for the diagnosis and treatment of major diseases.

#### Mitochondrial transplantation

Mitochondrial transplantation represents an innovative cellular therapy approach for treating major diseases, involving the isolation and transplantation of healthy and functionally active mitochondria into defective cells to replace impaired ones [315]. Animal experiments have demonstrated the efficacy of mitochondrial transplantation in various disease models, including cardiac arrest, cardiac ischemia–reperfusion injury, acute respiratory distress syndrome, PD, spinal cord injury, liver injury, kidney injury, and sepsis-induced multiple organ dysfunction [316–322]. Potential therapeutic effects of mitochondrial transplantation have also been observed in cases of sepsis-induced multiple organ dysfunction [33].

Moreover, multiple clinical studies have reported the application and effectiveness of mitochondrial transplantation. For instance, in 2013, Masuzawa et al. [323] pioneered the clinical application of autologous mitochondrial transplantation to alleviate myocardial ischemia–reperfusion injury following pediatric cardiac surgery. In 2021, Guariento et al. [324] additionally ascertained that autologous mitochondrial transplantation could aid children with ischemia–reperfusion injury after cardiac surgery using extracorporeal membrane oxygenation and improve their overall cardiac function. Furthermore, in 2018, Fang et al. [325] performed autologous mitochondrial transplantation in a patient experiencing repeated in vitro fertilization failure due to infertility and successfully achieved conception and delivery of a healthy male infant by the patient. In 2022, Jacoby et al. [326] transplanted healthy mitochondria derived from the mother of a child with mtDNA depletion syndrome into the child’s CD34^+^ hematopoietic stem cells to augment their mitochondria. The expanded hematopoietic stem cells were subsequently reinfused into the child, resulting in increased mtDNA content in peripheral blood cells, improved aerobic function, and increased the weight of the child. Additionally, this treatment exhibited favorable safety and tolerability.

Both preclinical and clinical studies have shown that mitochondrial transplantation is an advanced technology with significant therapeutic potential for treating various major diseases. However, its clinical application is hindered by several challenges including the necessity for further investigation into long-term safety, efficacy, and potential side effects. Despite ongoing optimization efforts which have enhanced success rates and reduced treatment risks considerably; it’s important to acknowledge the inherent limitations such as incomplete elimination of pathological mitochondria using current techniques or possible requirement for multiple transplants in some patients to achieve optimal therapeutic effects. Furthermore, issues related to batch extraction of mitochondria, their storage, host transplant rejection reactions, as well as ethical considerations remain to be addressed.

## Conclusions

In this review, we focused on mitochondrial quality control and provided a comprehensive overview of its fundamental characteristics. We framed this review by discussing three key aspects, namely mitochondrial morphology and dynamics, mitochondrial function and metabolism, and mitochondrial protein expression. Additionally, we analyzed the specific manifestations of mitochondrial quality imbalance in major diseases, including ischemic-hypoxic, inflammatory, viral, metabolic, and degenerative diseases and tumors, revealing their close connection with mitochondrial quality imbalance. Furthermore, we delved into novel therapeutic approaches targeting mitochondrial quality control, including small-molecule drugs that regulate key aspects of mitochondrial quality, nanomaterials that target mitochondria, and vesicle therapy and cellular treatment methods like mitochondrial transplantation as emerging options. This review offers a new perspective for understanding the common mechanisms underlying the development of major diseases while providing theoretical support and practical directions for the clinical application of new therapies in the intervention of major diseases.

The focus of mitochondrial research has transitioned from general inquiries about their damage extent to specific considerations regarding potential harm to pathways regulating mitochondrial quality control. A comparative analysis reveals substantial promise for translating advancements in mitochondrial quality control into clinical applications. Existing treatment modalities have demonstrated efficacy in select diseases; however, innovative strategies like gene editing, stem cell therapy, mitochondrial transplantation, and vesicle-based therapies offer prospects for precise intervention in mitochondrial function. This could enhance treatment outcomes and alleviate suffering among patients with prevalent conditions including heart disease, cancer, and neurodegenerative disorders. Yet, the implementation of these novel methods necessitates further investigation through rigorous research and clinical trials, to ensure their long-term safety and effectiveness.

The common patterns of mitochondrial quality imbalance in major diseases warrant further exploration, and the identification of more targeted intervention targets is imperative. Researchers can integrate advanced techniques, such as gene editing, to investigate effective strategies for targeted intervention of mitochondrial quality and develop combination therapy protocols for major diseases. Additionally, the advancement of diagnostic techniques, such as mitochondrial functional assessment and imaging, will facilitate early screening and monitoring of major diseases.

However, we currently encounter technological constraints that may hinder the clinical application and dissemination of research achievements in mitochondrial quality control. For example, despite emerging mitochondrial therapies, such as mitochondrial transplantation showing potential efficacy in preliminary research and clinical trials, there is still an incomplete understanding of the long-term safety and effectiveness of these methods. Technological bottlenecks related to the source of mitochondrial supply, preservation methods, and mitochondrial transplantation procedures directly impact the clinical application and dissemination of research outcomes. The translation of fundamental scientific research into clinical applications often necessitates a prolonged timeframe. Only by addressing these issues can mitochondrial therapeutic approaches be more widely used to improve patients’ quality of life and manage major diseases.

Overall, mitochondrial quality control plays a crucial role in the prevention and treatment of major diseases. Strategies for disease prevention and treatments targeting mitochondrial quality control hold promising prospects for delivering improved therapeutic options in clinical practice and enhancing patient health outcomes.

## Data Availability

Not applicable.

## Abbreviations

Aβ: Amyloid-β
AD: Alzheimer’s disease
ATF5: Activating transcription factor 5
BCL2: B-cell lymphoma 2
CLPXP: Caseinolytic protease X and protease P complex
CoQ: Coenzyme Q
COVID-19: Coronavirus disease 2019
CsA: Cyclosporin A
CYPD: Cyclophilin D
Cyt C: Cytochrome C
DAMPS: Damage-associated molecular patterns
DRP1: Dynamin-related protein 1
ETC: Electron transport chain
FIS1: Fission mitochondrial 1
FUNDC1: FUN14 domain containing 1
GR: Glucocorticoid receptor
HBV: Hepatitis B virus
HBX: Hepatitis B virus X protein
HCV: Hepatitis C virus
HIV: Human immunodeficiency virus
HSPD1: Heat shock protein family D member 1
IL: Interleukin
LONP1: Lon peptidase 1
LRP130: Leucine-rich protein 130 kD
MAD: Mitochondrial-associated degradation
MAVS: Mitochondrial antiviral-signaling protein
MCU: Mitochondrial calcium uniporter
MDV: Mitochondria-derived vesicle
Mdivi-1: Mitochondrial division inhibitor-1
MFN: Mitofusin
MMP: Mitochondrial membrane potential
mPTP: Mitochondrial permeability transition pore
MSCs: Mesenchymal stem cells
mtDNA: Mitochondrial DNA
mtUPR: Mitochondrial unfolded protein response
MTSs: Mitochondrial targeting signal peptides
NADH: Nicotinamide adenine dinucleotide hydrogen
NAFLD: Non-alcoholic fatty liver disease
NLRX1: NLR family member X1
OPA1: Optic atrophy 1
OXPHOS: Oxidative phosphorylation
p62: Ubiquitin-binding protein p62
Pae: Paeonol
PAH: Pulmonary arterial hypertension
PARKIN: Parkin RBR E3 ubiquitin-protein ligase
PD: Parkinson’s disease
PGC-1α: Peroxisome proliferator-activated receptor gamma coactivator-1α
PINK1: Phosphatase and tensin homolog-induced kinase 1
PROTACs: Proteolysis-targeting chimeras
ROS: Reactive oxygen species
S3: 15-Oxospiramilactone
SARS-CoV-2: Severe acute respiratory syndrome coronavirus 2
SKP2: S-phase kinase-associated protein 2
SOD2: Superoxide dismutase 2
SS: Szeto-schiller
TCA: Tricarboxylic acid
TEMPO: 2,2,6,6-tetramethylpiperidine-1-oxyl radical
TFAM: Mitochondrial transcription factor A
TOM: Translocase of outer mitochondrial membrane
TLR: Toll-like receptor 9
TNF-α: Tumor necrosis factor-α
TPP: Triphenylphosphonium
UCP: Uncoupling protein
UPS: Ubiquitin–proteasome system
VDAC: Voltage-dependent anion channel
